# KAT8-mediated epigenetic modifications: Physiological functions, heterogeneity in disease, and advances in targeted development

**DOI:** 10.1016/j.isci.2026.116680

**Published:** 2026-07-10

**Authors:** Zengjin Wang, Guanghui Ren, Peng Gao

**Affiliations:** 1 Medical Research Central, Shandong Provincial Key Medical and Health Laboratory of Translational Medicine in Microvascular Aging, The First Affiliated Hospital of Shandong First Medical University & Shandong Provincial Qianfoshan Hospital, Jinan 250014, Shandong, China; 2 Department of Pharmacy, The Second Qilu Hospital of Shandong University, Shandong University, 247 Beiyuan Street, Jinan 250033, Shandong, People's Republic of China

**Keywords:** KAT8, MOF/MYST1, histone acetyltransferase, H4K16ac, non-histone acetylation, epigenetic regulation, tumorigenic mechanisms, KAT8 inhibitors

## Abstract

KAT8 (MOF/MYST1) is a core histone acetyltransferase of the MYST family. Beyond its canonical H4K16ac activity, KAT8 catalyzes diverse acylations and regulates stem cell biology, DNA repair, metabolism, and immunity. This review systematically integrates KAT8’s regulatory networks across physiology and disease. We decipher the molecular basis of its context-dependent “double-edged sword” role in cancer, acting predominantly as an oncoprotein yet exhibiting tumor-suppressive functions under specific conditions. We evaluate current KAT8 inhibitor development, from early non-selective compounds to selective leads, and highlight persistent translational hurdles including insufficient specificity and limited *in vivo* efficacy. This work provides a comprehensive framework that clarifies recent controversies—such as whether H4K16ac primarily governs transcription or replication timing, and which KAT8-containing complex catalyzes, which acetylation mark—and establishes a rationale for future precision-targeting strategies and biomarker development grounded in KAT8 functional heterogeneity.

## Graphical abstract

**Figure:**
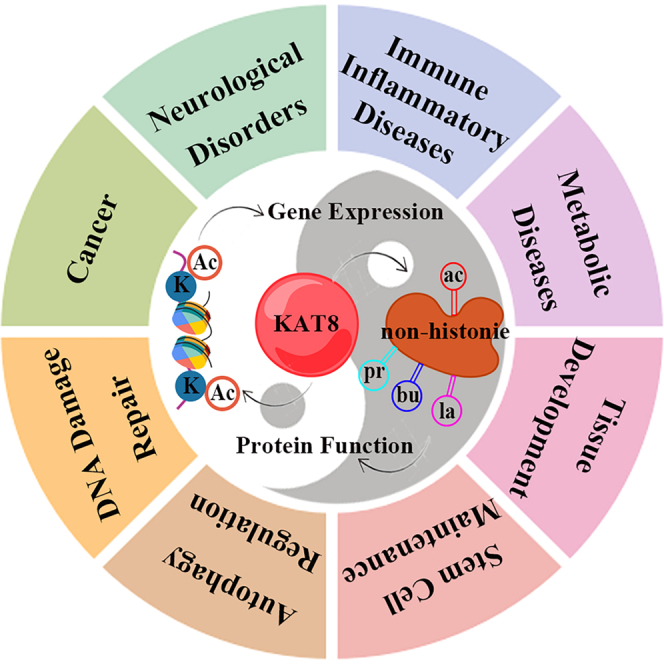

## Teaser

Immunology; Physiology; Epigenetics

## Introduction

**Figure 1:**
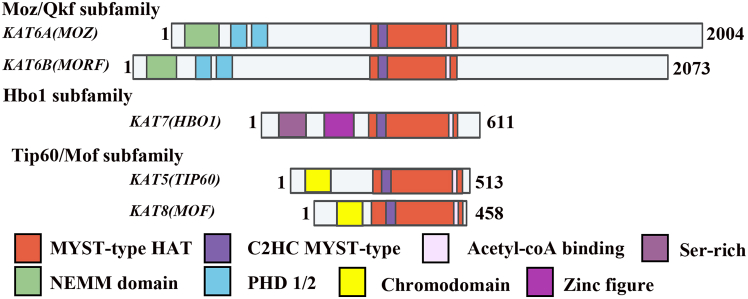
Domain architecture and 3D structure of human MYST family histone acetyltransferases HATs of the MYST family are classified into three distinct subfamilies, each defined by a conserved domain architecture. The Moz/Qkf subfamily comprises *KAT6A* (*MOZ*) and *KAT6B* (*MORF*); the HBO1 subfamily consists solely of *KAT7* (*HBO1*); and the Tip60/MOF subfamily includes *KAT5* (*TIP60*) and *KAT8* (*MOF*). The MYST domain serves as the core catalytic module across all family members, harboring an acetyl-CoA-binding region and a C2HC-type zinc finger. A chromodomain—a hallmark of *KAT5* and *KAT8*—mediates protein-nucleic acid interactions. PHD-type zinc fingers, characteristic of *KAT6A* and *KAT6B*, are thought to contribute to chromatin targeting. The N terminus of *KAT7* contains a serine-rich region and a zinc finger distinct from the canonical C2HC type. Additionally, *KAT6A* and *KAT6B* bear an N-terminal NEMN domain, whose function remains incompletely characterized; it is putatively involved in protein-protein interactions or autoregulation.

The MYST family is a highly conserved group of lysine acetyltransferases (KATs). The MYST family was initially defined through the identification of yeast SAS2 (Something About Silencing 2) and SAS3 (Something About Silencing 3) genes, which were found to be homologous to human MOZ (monocytic leukemia zinc finger) and TIP60 (HIV Tat-interacting protein); the family name MYST derives from its four founding members: MOZ, Ybf2/Sas3 (Yeast Bromodomain Factor 2/Something About Silencing 3), Sas2, and Tip60.^1^^,^^2^ The *in vivo* histone acetyltransferase (HAT) activity of the Drosophila homolog MOF (males absent on the first) was later demonstrated by Hilfiker et al., who showed that it specifically catalyzes H4K16 acetylation for dosage compensation.^3^ To make it easier to follow, we have listed the names of MYST family members across different species in Table S1. The human MYST family includes five members: KAT5 (TIP60), KAT6A (MOZ), KAT6B (MORF), KAT7 (HBO1), and KAT8 (MOF/MYST1).^4^^,^^5^ All share a conserved MYST domain with a C2HC zinc finger and an HAT catalytic domain^6^ (Figure 1). MYST members regulate transcription, development, stress responses, and hormone signaling via acetylation of histones and non-histone substrates, yet exhibit distinct substrate specificity.^7^ Several MYST paralogs (MOF, MOZ, HBO1) also have propionyltransferase activity, expanding their epigenetic reach beyond classical acetylation.^8^

KAT8 is one of the most versatile MYST members. It was subsequently validated as the core catalytic enzyme for X chromosome dosage compensation. This process remodels chromatin accessibility by specifically catalyzing H4K16ac. As a result, it globally upregulates transcription from the single male X chromosome.^3^^,^^9^^,^^10^ Global *Kat8* knockout in mice leads to embryonic lethality at the blastocyst stage, with failure to complete post-implantation development.^11^ This broad substrate portfolio positions KAT8 at the intersection of epigenetic regulation and intracellular signaling networks. Such convergence has profound implications for both normal physiology and disease pathogenesis.

Dysregulation of KAT8 is causally linked to human diseases. In cancer, KAT8 is frequently overexpressed in malignancies such as non-small cell lung cancer (NSCLC), esophageal squamous cell carcinoma (ESCC),^12^^,^^13^ and acute myeloid leukemia.^12^^,^^13^^,^^14^^,^^15^ In these malignancies, KAT8 drives proliferation, metabolic reprogramming, and therapeutic resistance. Conversely, loss of function mutations in KAT8 causes a neurodevelopmental disorder characterized by intellectual disability and epilepsy.^16^ Aberrant KAT8-mediated acylation (including lactylation and propionylation) contributes to psoriasis, rheumatoid arthritis, hepatic steatosis, and age-related vascular dysfunction and skin aging.^17^^,^^18^^,^^19^^,^^20^^,^^21^ These observations collectively establish KAT8 not merely as a chromatin modifier, but as a central node in disease-relevant signaling networks. Given this extensive pathological involvement, KAT8 has emerged as an attractive therapeutic target. Selective small-molecule inhibitors and proteolysis-targeting chimera (PROTAC)-based degraders are actively being developed. The ultimate goal is to exploit the enzyme’s context-dependent “double-edged sword” nature for precision oncology.

Recently, two studies have refined the canonical KAT8-H4K16ac model regarding complex-dependent substrate specificity and the role of H4K16ac in transcription versus replication timing.^22^^,^^23^ These findings are discussed in detail in “complex-dependent substrate selectivity” and “KAT8 in malignancy: a unified framework for context-dependent functions.” Throughout this review, we explicitly address these controversies and integrate them into a unified, context-driven framework. The following sections will systematically dissect the molecular architecture, physiological functions, and disease-specific roles of KAT8, ultimately framing a roadmap for translating KAT8 biology into clinical applications. To achieve this, we first dissect the molecular architecture of KAT8. In “structural basis of KAT8 functional divergence: complex-dependent substrate selectivity,” we address a fundamental principle: how KAT8 achieves its functional diversity through complex-dependent substrate selectivity.

## Structural basis of KAT8 functional divergence: Complex-dependent substrate selectivity

### Enzymatic properties of KAT8

**Figure 2:**
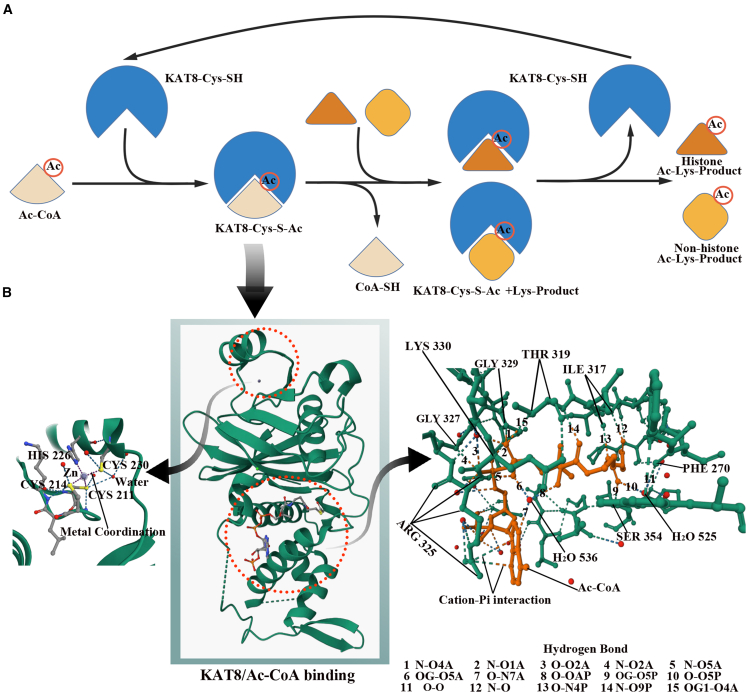
Ping-pong mechanism of histone acetylation catalyzed by KAT8 and its structural interactions with acetyl-coenzyme A Schematic of KAT8’s ping-pong catalytic mechanism for histone acetylation. (A) It involves two steps: first, Ac-CoA binds KAT8’s active site, transferring its acetyl group to Cys316 to form a covalent acetyl-enzyme intermediate (KAT8-Cys-S-Ac) with CoA-SH release. Then, the ε-amino group of histone target lysine (e.g., H4) attacks the intermediate, accepting the acetyl group to form acetylated histone and regenerate free Cys316, completing the cycle. (B) Detailed structural interactions between KAT8 and Ac-CoA, based on the crystal structure PDB: 2GIV.^24^ The structural model illustrates Ac-CoA docking into the catalytic pocket of KAT8 and the extensive interaction network formed with key amino acid residues. Metal coordination: a zinc ion (Zn^2+^) is tetrahedrally coordinated by four conserved residues of KAT8—Cys211, Cys214, His226, and Cys230-forming a stable metal coordination center that is essential for maintaining the structural conformation of the active pocket. Ac-CoA binding and stabilization: the Ac-CoA molecule establishes an extensive network of hydrogen bonds with multiple KAT8 residues (including Thr319, Ile317, Phe270, and Ser354) via its phosphate groups and adenosine moiety, as indicated by numbered contacts in the panel. The carbonyl oxygen of the acetyl group forms a hydrogen bond with a structural water molecule (H_2_O 525). Additionally, cation-π interactions likely occur between the guanidinium group of arginine residues (e.g., Arg325) and the adenosine ring of Ac-CoA, collectively anchoring and properly orienting Ac-CoA within the binding pocket. Numbers 1–15 denote individual hydrogen bonds formed between Ac-CoA and amino acid residues or water molecules of KAT8, where hydrogen bond donors are contributed by KAT8 or bound water molecules, and acceptors are provided by Ac-CoA.

KAT8 follows a ping-pong mechanism typical of bisubstrate enzymes. First, it binds acetyl-coenzyme A (Ac-CoA) to form an acetylated enzyme intermediate with release of coenzyme A. Then, histone H4 binds, and the acetyl group is transferred to H4K16 (Figure 2A). Its catalytic function depends on a conserved HAT domain containing a zinc finger and the key residue Glu350^25^^,^^26^ (Figure 2B). Its primary product is H4K16ac, with progressive acetylation of H4K5, K8, and K12 observed upon prolonged incubation.^27^^,^^28^

KAT8 activity is tightly controlled by autoacetylation of K274 within the MYST domain. Structural studies show that the acetylated Lys-274 side chain flips into the active site. In the unacetylated state, the active site adopts an inactive conformation that is unfavorable for substrate binding.^7^^,^^29^ The deacetylase sirtuin 1 (SIRT1) deacetylates K274, reducing KAT8 activity and promoting its ubiquitin-mediated degradation.^30^ Mutagenesis confirms this requirement: K274R abolishes activity, while the K274Q mimic retains only ∼2.9%.^31^ It is worth noting that K-to-Q mutations are commonly used to mimic acetylated lysine, but glutamine does not fully recapitulate the structural and electrostatic properties of acetyl-lysine. The observation that K274Q retains only ∼2.9% activity compared to wild-type KAT8 should be interpreted with caution, as this may reflect both the importance of the acetyl group and the imperfect nature of the mimetic. Structural studies using acetyl-lysine analogs or direct detection of acetylated K274 would provide more definitive evidence.

### Complex-dependent substrate selectivity

**Figure 3:**
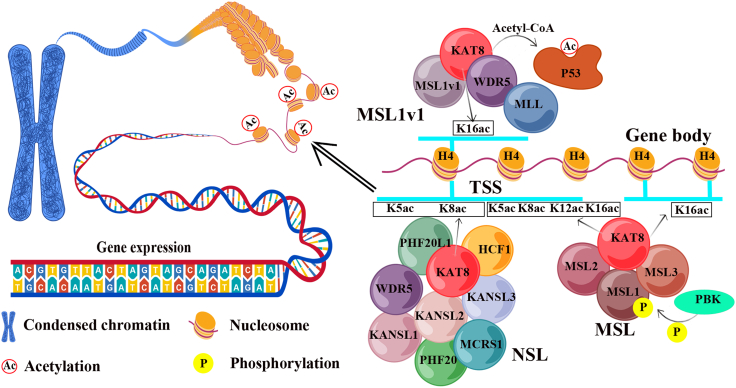
Schematic of the role of KAT8-mediated H4K16ac in chromatin regulation and transcription This schematic illustrates the distribution and mechanism by which the histone acetyltransferase KAT8 exerts its functions through two distinct complexes, NSL and MSL, across different genomic regions. Transcription start site (TSS) region: the NSL complex (comprising KAT8, KANSL1-3, MCRS1, PHF20, WDR5, HCF1, and other components) localizes to promoter regions. It catalyzes multi-site acetylation of histone H4 at lysines 5, 8, and 12, facilitating RNA polymerase II recruitment and basal transcription. Gene body region: The MSL complex (composed of KAT8, MSL1, MSL2, and MSL3) is enriched in gene bodies. It is primarily responsible for catalyzing H4K16ac to promote transcriptional elongation and maintain an open chromatin state. PBK-mediated phosphorylation of MSL1 enhances the assembly and stability of this complex. H4K16ac collectively promotes an open chromatin state and gene expression by reducing the affinity between histones and DNA, inhibiting tight chromatin compaction, and other mechanisms. MSL1V1, WDR5, MLL, and KAT8 form a complex that, while catalyzing H4K16ac, is mainly responsible for the acetylation of non-histone proteins.

KAT8 functions through two evolutionarily conserved multi-protein assemblies: the male-specific lethal (MSL) complex and the non-specific lethal (NSL) complex,^32^ which differ markedly in substrate specificity and biological function (Figure 3). In contrast to earlier models, the NSL complex does not catalyze H4K16ac to a substantial degree in human somatic cells; instead, H4K16ac is deposited almost exclusively by the MSL complex. The NSL complex primarily catalyzes H4K5ac and H4K8ac at promoter regions of housekeeping genes.^22^

The MSL complex displays strict substrate specificity for H4K16ac. It is composed of KAT8, MSL1, MSL2, and MSL3.^33^^,^^34^^,^^35^ Crucially, the MORF4-related gene (MRG) domain of MSL3 is required for maintaining the MSL complex stability. Through this structural engagement, the complex inhibits the binding of chromatin remodelers such as imitation switch (ISWI) and specifically deposits H4K16ac. This maintains an open chromatin state that is conducive to transcriptional elongation. Knockdown of core components (e.g., MSL1) markedly reduces cellular H4K16ac without affecting other histone acetylation sites, while KAT8 knockout causes near-complete loss of H4K16ac and aberrant chromatin compaction.^36^^,^^37^^,^^38^ This observation suggests evolutionary specialization of MSL complex substrate specificity. In mammalian cells, the MSL complex is enriched across gene bodies, likely contributing to transcriptional elongation and associated with open chromatin states.^22^^,^^39^ In embryonic stem cells, MSL complex-driven H4K16ac ensures proper developmental gene regulation; in DNA repair, it creates an open chromatin environment that recruits key repair factors.^22^^,^^32^^,^^35^ Pathologically, this MSL “vehicle” can be hijacked. In NSCLC, its strict H4K16ac activity at the *S-phase kinase-associated protein 2* (*SKP2*), promoter accelerates S-phase progression. In tumor immunity, it drives *programmed death ligand 1* (*PD-L1*) expression to facilitate immune escape.^14^^,^^40^ Thus, the MRG-keyed engine promotes oncogenesis through specific gene body targeting.

In contrast, the NSL complex contains at least nine core subunits, including KAT8, KAT8 regulatory NSL complex subunit 1 (KANSL1, an MSL1 homolog), KANSL2, KANSL3, microspherule protein 1 (MCRS1), Plant homeodomain (PHD) finger protein 20 (PHF20), PHD finger protein 20-like 1 (PHF20L1), host cell factor C1 (HCFC1), and WD repeat domain 5 (WDR5).^22^ KANSL1 binds and activates KAT8 via its NSL1 HAT activation motif (NHAM) domain, complementary to the MRG domain of MSL3.^34^ This engages KAT8 for a different destination: the promoters of housekeeping genes. The NSL complex has a “broad-spectrum” payload, but as rigorously demonstrated by Radzisheuskaya et al.,^22^ its primary *in vivo* substrates are H4K5ac and H4K8ac, not H4K16ac. Quantitative chromatin immunoprecipitation sequence (ChIP-seq) and mass spectrometry analyses revealed that NSL complex depletion does not reduce global H4K16ac levels. MSL complex disruption abolishes the vast majority of H4K16ac. The NSL complex does catalyze multi-site acetylation (H4K5/8/16ac) *in vitro*, but this broad activity is not recapitulated *in vivo*. This broad acetylation, often in cross-talk with mixed-lineage leukemia (MLL) complexes that deposit H3K4me2, recruits RNA polymerase II and sustains basal transcription.^22^^,^^34^^,^^41^ NSL activity is directly linked to cellular metabolism, as KAT8 requires acetyl-CoA as its donor; metabolic shifts therefore modulate NSL-mediated acetylation and basal transcription.^22^^,^^42^ Furthermore, the NSL complex has been shown to regulate mitochondrial gene expression, extending its regulatory reach beyond nuclear chromatin and allowing it to sense and respond to changes in cellular energy homeostasis.^43^ Collectively, all these mechanisms enable the NSL complex to integrate diverse intracellular signals into precise chromatin-based transcriptional outcomes, thereby sustaining housekeeping gene expression and adapting to changing microenvironments.

A note on interpretation: earlier studies using purified NSL complex components reported *in vitro* H4K16ac activity, leading to the widespread assumption that the NSL complex contributes to cellular H4K16ac. However, the work of Radzisheuskaya et al.^22^ convincingly shows that in living human cells, H4K16ac is not dependent on NSL complex integrity. This discrepancy highlights the importance of validating biochemical activities with context-matched *in vivo* assays and underscores a central theme of this review: KAT8’s functional output is dictated not by its catalytic domain alone, but by its associated protein complex and cellular environment.

Ultimately, the dichotomy between the MSL and NSL complexes illustrates that MSL3 and KANSL1 play important roles as key regulatory factors in KAT8-driven epigenetic regulation. The MRG domain key builds a vehicle optimized for deep penetration into gene bodies with a single, precise modification (H4K16ac) to regulate elongation and repair, while the NHAM domain key builds a vehicle optimized for promoter anchoring with a broad, multi-site modification profile (H4K5/8/16ac) to maintain cellular homeostasis. This structural division of labor allows a single enzyme to act as both a tumor suppressor (via NSL-mediated target of methylation-induced silencing 1 (TMS1) maintenance) and an oncogene (via MSL-mediated SKP2 or PD-L1 activation) depending entirely on which key is inserted.

A note on interpretation: earlier studies using purified NSL complex components reported *in vitro* H4K16ac activity, leading to the assumption that NSL contributes to cellular H4K16ac. However, Radzisheuskaya et al.^22^ convincingly show that in living human cells, H4K16ac is not dependent on NSL complex integrity. This discrepancy highlights the importance of validating biochemical activities with context-matched *in vivo* assays and underscores a central theme of this review: KAT8’s functional output is dictated not by its catalytic domain alone, but by its associated protein complex and cellular environment.

### H4K16 acetylation: A molecular switch for chromatin remodeling

KAT8 governs chromatin architecture and gene expression by catalyzing H4K16ac. Structural studies have established that H4K16ac serves as a unique molecular switch for chromatin decompaction, operating through distinct mechanisms. First, by neutralizing the positive charge on the histone H4 tail, H4K16ac reduces histone DNA affinity, destabilizing the 30 nm fiber and disrupting internucleosomal interactions, functionally equivalent to deleting the entire H4 tail.^35^^,^^44^ Consequently, H4K16ac actively impedes the ability of chromatin to form higher-order cross-fiber interactions, effectively “locking” the chromatin in an open conformation. Second, H4K16ac overrides the higher order folding normally induced by linker histone H1.^45^ Third, by disrupting the H4 tail-H2A interface required for condensed structures, H4K16ac inhibits ATP-dependent remodelers like ATP-utilizing chromatin assembly and remodeling factor (ACF)/ISWI from compacting nucleosomes.^44^^,^^46^^,^^47^ KAT8 depletion results in complete loss of H4K16ac and aberrant chromatin condensation, an early structural defect that drives developmental arrest and apoptosis.^38^ In embryos lacking H3.3 or KAT8, reduced H4K16ac coincides with aberrant H1 accumulation and chromosome segregation errors; concurrent H1 knockdown partially rescues this phenotype, confirming the H4K16ac-H1 axis in chromatin structural maintenance.^48^ This acetylation mark activates chromatin structure either by MSL-triggered opening of gene bodies or by NSL-associated promoter priming, and thus translates metabolic and signaling cues into diverse transcriptional outcomes.^49^ For gene regulation, H4K16ac provides a structural platform for transcription factor and cofactor recruitment. In mammalian X chromosome dosage compensation, KAT8 accumulates at active gene promoters, and H4K16ac facilitates RNA polymerase II recruitment and transcriptional initiation.^50^ In mouse embryonic stem cells, H4K16ac is specifically enriched at transcription start sites and enhancers of active genes. Importantly, H4K16ac defines a novel class of active enhancers marked by H3K4me1 and H4K16ac but lacking canonical H3K27ac, with independent transcriptional activation capacity, indicating that MOF marks a distinct enhancer subset from those regulated by p300/CREB-binding protein (CBP).^51^ For specific loci such as TMS1/apoptosis-associated speck-like protein containing a CARD (ASC), H4K16ac is precisely deposited at nucleosomes flanking CpG islands, fundamental to maintaining nucleosome positioning and active gene expression; KAT8 downregulation causes loss of H4K16ac, disordered nucleosome positioning, and gene silencing.^52^

This biophysical “opening” of local chromatin architecture constitutes the primary mechanism by which KAT8 facilitates transcriptional activation, a principle that underlies its function in stem cell pluripotency and DNA damage repair (DDR) discussed in subsequent sections.

### KAT8-mediated non-histone post-translational modifications

In addition to the canonical MSL and NSL complexes, a distinct KAT8-containing complex was reported by Li et al.^53^ KAT8 forms a distinct complex with male specific lethal 1 variant 1 (MSL1v1), WD repeat-containing protein 5 (WDR5), and MLL^35^ (Figure 3). This complex comprises KAT8 and an alternative splicing variant of MSL1, originally termed MSL1v1 (also known as KIAA1267 or LOC284058). Subsequent studies have shown that MSL1v1 is actually the same protein as KANSL1, which is also a core component of the NSL complex.^54^ However, the complex described by Li et al.^53^ is distinct from the canonical NSL complex in subunit composition and substrate specificity. To avoid confusion, we refer to this assembly as the KAT8-MSL1v1 (or KAT8-KANSL1) complex. To distinguish naming conventions for cross species KAT8/MOF genes, proteins, and complexes, we have clarified this nomenclature in Table S2. KAT8-MSL1v1 complex primarily acetylates non-histone substrates such as p53 at K120, thereby promoting apoptosis, and is not responsible for bulk H4K16 acetylation.^53^ Importantly, later studies have shown that some substrates previously attributed to this complex (e.g., LSD1) are actually acetylated by the NSL complex in a KAT8-dependent manner.

KAT8 senses changes in cellular metabolism and then converts these signals into non-histone acetylation and diverse new acylation modifications. It acetylates diverse non-histone substrates: p53 (K120) to promote apoptosis, interferon regulatory factor 1 (IRF1) (K78) to drive phase separation, and AR/AR-v7 to regulate stability and transcription.^40^^,^^55^^,^^56^ Substrate choice of KAT8 depends on the local microenvironment and metabolite levels. In neurodevelopment, MOF acts as a propionyltransferase. By responding to metabolic signals like propionyl-CoA, MOF then balances H4K16 acetylation and propionylation to control the proliferation and differentiation of neural stem and progenitor cells.^16^ In tumors with active metabolic reprogramming, KAT8 acts as a protein acetyltransferase. In colorectal cancer (CRC) models, it lactylates eEF1A2 to drive tumor-specific protein synthesis.^12^ In fibroblast fibrosis models, KAT8 lactylates latent transforming growth factor β binding protein 1 (LTBP1) at K752 to increase collagen synthesis.^57^

It is important to note that KANSL1 is also an integral subunit of the NSL complex. The KAT8-KANSL1 complex described by Li et al.^53^ may represent a sub-complex or a distinct assembly that differs from the full NSL complex. Given the current literature, we recommend that future studies use precise complex nomenclature (e.g., “NSL complex” for the nine-subunit assembly, and “KAT8-KANSL1 or KAT8-MSL1v1 complex” for the binary or smaller complex) to avoid confusion.

In summary, KAT8 serves as a key hub that maps complex metabolic signals (such as propionylation and lactylation) directly onto non-histone substrates. By dynamically regulating protein stability, phase separation, and transcription networks, KAT8 directs cell fate across different cellular environments and disease models. KAT8-mediated non-histone acetylation and other acylations are summarized in Table 1. This structural basis explains the functional duality of KAT8, enabling it to shift from nuclear epigenetic control to the regulation of whole-cell networks.

**Table 1:** Summary of KAT8 protein interactions

Interacting protein	Interaction region	Modification site	Physiological/pathological state/disease model	Functional consequence post-interaction	Reference
**I. KAT8 Modifying other proteins (acetylation)**					

1. Histone acetylation					
Histone H4	MSL/NSL complexes	H4K5/K8/K16	broad cellular processes (EMT, cell cycle, DNA repair)	regulates transcription, chromatin structure, DNA repair, cell fate; impacts EMT and tumor progression	Wei et al.^17^; Xiang et al.^39^
Histone H3	not specified	H3K27	WSSV-infected shrimp	enhances transcriptional activity of antiviral genes	Lv et al.^58^
2. Acetylation of transcription factors and regulatory proteins					
p53	MYST domain; MSL1v1 recruits	K120	DNA damage; cancer (lung, liver)	enhances p53 pro-apoptotic gene activation, promotes apoptosis	Singh et al.^40^; Li et al.^53^; Chang et al.^59^
c-Myc	HAT domain binds c-Myc DBD	K445	esophageal squamous cell carcinoma (ESCC)	stabilizes c-Myc, upregulates target genes, promotes proliferation	Zhang et al.^12^
FSCN1 (Fascin)	MYST domain binds Fascin β2-domain	K41, K241	ESCC	inhibits F-actin bundling, reduces filopodia/invadopodia, suppresses migration/invasion; low acetylation correlates with poor prognosis	Li et al.^60^
IRF1	LLPS with KAT8 under IFNγ	K78	tumor immune evasion; interferon regulation	enhances DNA binding, forms condensates activating PD-L1 expression	Wu et al.^55^
IRF3	MYST domain binds C terminus	K359	viral infection (VSV, SeV, HSV-1)	inhibits IRF3 recruitment to IFN promoters, suppresses interferon production	Huai et al.^61^
IRF7	MYST domain binds	lysine(s) not specified	viral infection	inhibits IRF7 binding to ISRE, reduces IFN1 expression	Li et al.^62^
PAX7	direct interaction (IP)	K105, K193	muscle stem cell self-renewal/differentiation	enhances PAX7 binding to target genes, regulates stem cell fate	Sincennes et al.^63^
ERα (estrogen receptor α)	recruited by PHF20 (via ERα K235me2)	K266, K268, K299	ER + breast cancer; liver cancer	promotes local H4K16ac and ERα target transcription (breast); stabilizes ERα, tumor-suppressive (liver)	Zhang et al.^64^; Wei et al.^65^
FOXO	direct interaction and activation	K180, K183	drosophila autophagy; shrimp antiviral immunity	forms positive feedback loop: FOXO activates KAT8, KAT8 acetylates FOXO, promoting autophagy	Liu et al.^10^; Lv et al.^58^
NRF2	direct interaction	K588	mycobacterium tuberculosis infection; NSCLC	enhances NRF2 nuclear retention/stability, activates antioxidant genes; promotes ferroptosis (TB) or stress adaptation (NSCLC)	Singh et al.^40^; Satish et al.^66^; Chen et al.^67^
3. Acetylation of enzymes and metabolism-related proteins					
KDM1A	binding not specified	K117	hepatocellular carcinoma (HCC)	promotes cytoplasmic localization and stability, enhances demethylase activity toward FKBP8, promotes survival and sorafenib resistance	Lv et al.^68^
LSD1	indirect via NSL complex	K432, K433, K436	EMT and tumor models	weakens nucleosome binding, inhibits H3K4 demethylation, suppresses EMT and invasion	Luo et al.^69^
PCK2	direct binding (IP)	K491	sorafenib-resistant HCC	stabilizes PCK2, inhibits degradation, promotes mitochondrial gluconeogenesis and resistance	Jing et al.^70^
PCK1	direct binding (IP)	K473	sorafenib-resistant HCC	promotes PCK1 degradation, forms “see-saw” switch with PCK2, synergizes in metabolic reprogramming and resistance	Jing et al.^70^
ATP5B	direct interaction	K201	heart failure	impairs ATP synthase, reduces ATP synthesis and mitochondrial respiration	Hu et al.^71^
AURKB	via MSL complex	K215	breast cancer	prevents ubiquitination/degradation, stabilizes protein, enhances kinase activity, promotes proliferation and c-Myc accumulation	Miao et al.^72^
YEATS4	likely via NSL complex	K64, K65, K69	bladder cancer	inhibits HUWE1-mediated ubiquitination/degradation, maintains genomic stability and proliferation	Xie et al.^56^
WSTF	direct interaction	K426	lung cancer cisplatin resistance	enhances WSTF activity, promotes DNA damage repair (HDR), inhibits apoptosis, enhances resistance	Sui et al.^73^
FASN	direct interaction (Co-IP)	lysine(s) not specified	various tumor cell lines	promotes FASN binding to TRIM21, leading to ubiquitination and degradation, inhibits lipogenesis and tumor growth	Lin et al.^74^
4. Acetylation of other structural and signaling proteins					
SEPP1	not specified	K247, K249	pancreatic cancer	increases stability and secretion, enhances binding to LRP8, inhibits MDSCs, activates CD8^+^ T cell immunity	Zhu et al.^75^
IL-33	predicted via HAT domain	lysine(s) not specified	allergic asthma	enhances stability, reduces degradation, exacerbates airway inflammation; inhibition alleviates asthma	Liu et al.^76^
Lamin A/C	interaction region not detailed	K311	nuclear envelope stability	maintains lamin stability and nuclear envelope integrity; deficiency causes abnormalities	Karoutas et al.^77^
RSF1	not mentioned	K1050	skin aging	promotes chromatin assembly/stability, maintains heterochromatin; reduced acetylation promotes senescence	Wang et al.^20^
SIRT6	direct interaction	K128, K160, K267	regulation of EMT and metastasis	inhibits SIRT6 deacetylase activity, relieves suppression of ZEB2, promotes EMT	Zhao et al.^78^
BRM	direct interaction	lysine(s) not specified	BRM+ lung cancer	Inactivates BRM protein	Kahali et al.^79^
TIP5 (NoRC)	binds near TAM domain	K633 (human K649)	late S-phase; glucose deprivation	reduces pRNA binding, promotes NoRC dissociation, leading to transcriptional silencing	Zhou et al.^80^
HPV E7	direct binding (CoIP)	lysine(s) not specified	HPV+ cervical cancer	enhances E7 stability/function, promotes pRb binding, releases E2F1, drives cell cycle and proliferation	Xu et al.^81^

**II. KAT8 modifying other proteins (lactylation)**					

1. Histone lactylation					
Histone H3	direct interaction	H3K9	rheumatoid arthritis (RA)	promotes pro-inflammatory gene (e.g., IL-6) expression, exacerbating joint inflammation	Dai et al.^18^
Histone H4	not mentioned	H4K12	macrophages (PLLA stimulation); skin aging	cooperates with KAT5 to promote H4K12la, enhances TGF-β1/β3 transcription, stimulates collagen synthesis	Zou et al.^82^
2. Non-histone lactylation					
eEF1A2	interaction not detailed	K408	colorectal cancer metabolic reprogramming	activates tumor-specific protein synthesis, drives proliferation in high-lactate microenvironment	Zhang; Xie et al.^12^^,^^83^
LTBP1	direct interaction (IP)	K752	skin aging model	promotes collagen I/III synthesis, aids skin regeneration	Zou et al.^57^
PCK2	direct interaction	K100	hyperlactatemia; liver ischemia-reperfusion	enhances PCK2 kinase activity, inhibits OXSM ubiquitination, promotes mitochondrial fatty acid synthesis, exacerbating oxidative stress and ferroptosis	Yuan et al.^84^
MDH2	direct interaction (IP)	K239	renal cell carcinoma (RCC)	enhances MDH2 activity, increases NADH/NAD^+^ and ATP, promotes NADPH generation and oxidative stress resistance, drives RCC progression	Tang et al.^85^

**III. KAT8 modifying other proteins (other acylations)**					

ALR	direct interaction (IP)	crotonylation, K78	MASLD	enhances ALR-MFN2 interaction, maintains MAM stability, improves mitochondrial function, reduces steatosis	Wang et al.^19^
HSP90	direct interaction (IP)	butyrylation, K754	ESCC 5-FU resistance	stabilizes HSP90, activates AKT signaling, promotes chemoresistance	He et al.^86^

**IV. Other proteins modifying KAT8**					

ATM	binds via ATM leucine zipper and KAT8 chromodomain	phosphorylation, T392	DNA damage repair; cell cycle checkpoint	positive feedback: KAT8 promotes ATM activation; ATM phosphorylates KAT8 to dissociate KAT8-53BP1, favoring HR over NHEJ	Singh et al.^40^; Gupta et al.^87^
SIRT1	direct interaction	deacetylation, K274	regulation of KAT8 activity; autophagy; cancer, aging	reduces KAT8 HAT activity and promotes degradation; negative feedback; In autophagy, deacetylates H4K16ac to relieve inhibition	Peng et al.^30^; Xu et al.^88^; Lu et al.^89^
MDM2	E3 ligase interaction	ubiquitination (site not specified)	HCC; breast cancer; notch activation	promotes KAT8 ubiquitination and degradation, decreases H4K16ac, enhances tumor proliferation/migration	Liu et al.^90^
USP10	binds KAT8 HAT domain via USP10 N terminus	deubiquitination, K410	ESCC	stabilizes KAT8, prevents degradation, increases nuclear accumulation and pro-tumorigenic functions	Li et al.^13^

**V. Scaffold and auxiliary proteins (no modification)**					

MSL1	binds via MBM to KAT8 PEHE segment	none (scaffold)	MSL complex assembly; dosage compensation; pluripotency	scaffold for MSL complex assembly, specifies H4K16ac catalysis	Badeaux et al.^33^; Li et al.^35^; Wang et al.^91^
MSL3	indirect via MSL1; MRG domain important	none (auxiliary)	MSL complex assembly/function	essential for efficient nucleosomal H4 acetylation by KAT8	Huang et al.^34^; Li et al.^35^
NSL1 (KANSL1)	binds via MBM to KAT8 NHAM region	none (scaffold/activator)	NSL complex assembly; basal transcription; neurodevelopmental disorders	NSL complex scaffold; NHAM activates KAT8 nucleosomal activity	Radzisheuskaya et al.^22^; Huang et al.^34^; Koolen et al.^92^
PHF20	NSL complex component	none (recruitment)	NSL function; ERα activation	tudor domain recognizes ERα K235me2, recruits KAT8 complex to promoters	Radzisheuskaya et al.^22^; Zhang et al.^64^
WDR5	shared subunit in NSL and MLL complexes	none (bridging)	NSL-MLL co-regulation; stress response (MSL1v1)	couples KAT8 and MLL activities; enhances H3K4 methylation; involved in non-histone acetylation in MSL1v1 complex	Radzisheuskaya et al.^22^; Li et al.^53^
MSL1v1 (MSL1 variant)	directly binds p53 (aa 705–800)	none (substrate recruitment)	DNA damage; p53 apoptosis	recruits p53 for KAT8 acetylation at K120, promoting pro-apoptotic transcription	Li et al.^35^^,^^53^

## KAT8 as a master regulator of cellular homeostasis

### The roles of KAT8 in stem cell regulation

KAT8 is widely implicated in stem cell self-renewal, cell cycle progression, DDR, neural development, and oxidative stress response.

**Figure 4:**
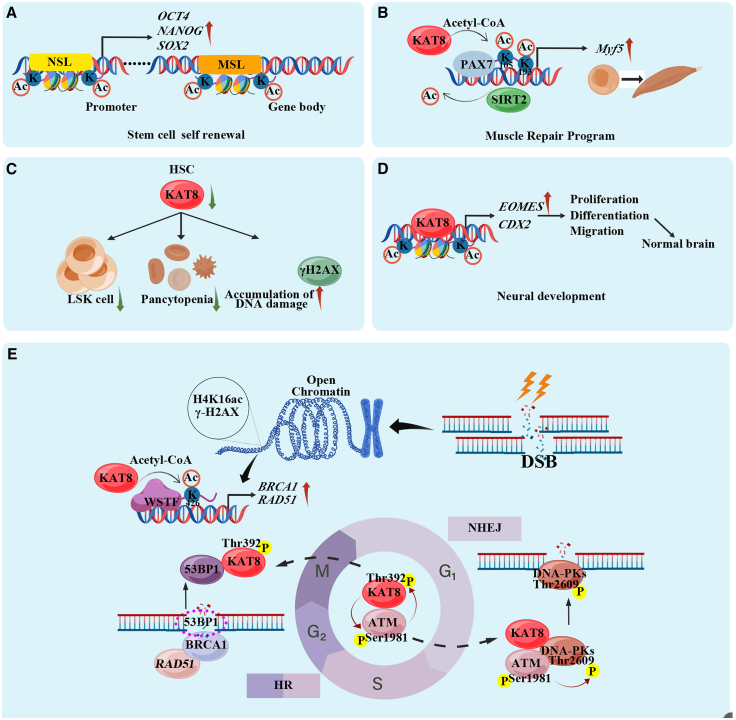
Multifaceted roles of KAT8 in stem cell maintenance, tissue development, and DNA repair (A) KAT8 maintains pluripotency: the NSL complex sustains pluripotency gene expression (e.g., *OCT4, NANOG*), while the MSL complex catalyzes H4K16ac to open chromatin and facilitate transcriptional elongation. (B) In muscle satellite cells, KAT8 acetylates PAX7 to enhance Myf5 binding, balancing self-renewal and differentiation. This is antagonized by SIRT2 and coupled to cellular metabolism. (C) KAT8 is essential for adult HSCs maintenance; its loss causes pancytopenia, reduced LSK cells, DNA damage, and impaired colony formation by regulating transcriptional programs with MLL1. (D) During neural development, KAT8-mediated H4K16ac opens chromatin and activates neurodevelopmental genes (e.g., *CDX2*). KAT8 deficiency leads to stem cell depletion, apoptosis, and severe brain malformation. (E) In the DNA damage response, KAT8-mediated H4K16ac relaxes chromatin and recruits MDC1 via the H2A.X acidic pocket, creating a platform for repair factors. During S/G2 phases, KAT8 interacts with ATM to promote its activation, causing 53BP1 dissociation and promoting BRCA1-mediated HR. In tumors, KAT8 acetylates WSTF (K426) to upregulate BRCA1/RAD51, causing cisplatin resistance. In G1 phases, this ATM phosphorylation event is attenuated, resulting in the stable retention of 53BP1 and directing repair toward NHEJ. Furthermore, KAT8 interacts with DNA-PKcs to modulate its phosphorylation, thereby influencing NHEJ efficiency.

In stem cell biology, KAT8 employs NSL and MSL complexes to govern pluripotency through spatially distinct mechanisms. The MSL complex occupies bivalent developmental genes, keeping them poised for rapid activation upon differentiation.^93^ (Figure 4A). This core mechanism exhibits highly specific molecular adaptations across different stem cell niches. In muscle stem cells, KAT8 acetylates PAX7 at K105/K193. This modification structurally modulates the PAX7 homeodomain interface, enhancing its binding affinity to target gene promoters like *Myf5*. SIRT2 dynamically antagonizes this process, coupling metabolic flux to muscle regeneration^63^ (Figure 4B).

In the hematopoietic system, KAT8 forms a localized epigenetic hub with the MLL1 complex, sustaining local H4K16ac to resist SIRT1. Disrupting this balance depletes hematopoietic stem cells (HSCs) and causes DNA damage^94^^,^^95^ (Figure 4C). During neural development, KAT8-catalyzed H4K16ac drives chromatin relaxation and activates the transcriptional program of key neural developmental genes, including caudal type homeobox 2 (CDX2).^96^ This balances neural stem/progenitor cells (NSPCs) proliferation and differentiation. Brain-specific knockout of KAT8 abolishes H4K16ac, triggering NSPC pool depletion, increased apoptosis, and abnormal cortical stratification, ultimately leading to cerebral hypoplasia. Human genetic evidence further confirms that loss-of-function mutations in KAT8 impair its acetyltransferase activity, causing intellectual disability, epilepsy, and related syndromes, establishing the central role of the KAT8-H4K16ac axis in neural development at the molecular, cellular, and clinical levels^16^^,^^96^ (Figure 4D).

In summary, KAT8 maintains stem cell function by responding to diverse signals: it prepares genes for rapid activation in embryonic stem cells, couples metabolism to regeneration in muscle stem cells, and balances antagonistic signals in HSCs. KAT8 thus acts as a central signal integrator, translating metabolic and differentiation cues into precise epigenetic changes to sustain stem cell homeostasis.

### KAT8 in cell cycle and DDR

KAT8-catalyzed H4K16ac is indispensable for cell cycle progression and genome stability. Loss of MSL or NSL complex components reduces global H4K16ac, inducing elevated replication stress, aberrant single-stranded DNA (ssDNA) accumulation, and enhanced replication stress signaling (e.g., phosphorylated replication protein A [pRPA], phosphorylated checkpoint kinase 1 [pCHK1]). These events ultimately lead to chromosomal fragility, breaks, and micronucleus formation, exacerbating genomic instability and perturbing cell cycle progression.^39^^,^^97^ KAT8 activity is feedback-regulated by the cell cycle and energy status: for instance, MOF-mediated acetylation of TIP5 (a subunit of the nucleolar remodeling complex, NoRC) at K633 is cell cycle-dependent, and TIP5 is deacetylated by SIRT1 under glucose deprivation. This enhances TIP5 binding to promoter RNA and promotes ribosomal DNA (rDNA) silencing, coupling metabolism to epigenetic regulation.^80^ *Kat8* deletion impairs replication fork stability and restart; cells exhibit slowed fork progression, increased fork stalling, and aberrant activation upon hydroxyurea (HU) or aphidicolin (APH). In germ cells, *Kat8* deficiency disrupts meiotic sex chromosome inactivation (MSCI), impairing timely removal of Radiation Sensitive 51 (RAD51)/DNA meiotic recombinase 1 (DMC1) and crossover formation.^98^

In the DDR, KAT8 relaxes chromatin structure via H4K16ac and interacts with ataxia telangiectasia mutated (ATM) kinase to create a repair-permissive chromatin environment. H4K16ac weakens internucleosomal interactions and promotes chromatin accessibility. KAT8 depletion or inactivation reduces H4K16ac, impairing foci formation and retention of repair factors (e.g., γH2AX, mediator of DNA damage checkpoint 1 (MDC1), tumor protein p53-binding protein 1 (53BP1), breast cancer type 1 susceptibility protein (BRCA1), RAD51) at DNA damage sites.^32^^,^^35^^,^^98^^,^^99^ H4K16ac mediates *trans*-nucleosomal interactions with the acidic pocket of H2A.X, providing a structural basis for MDC1 recruitment and subsequent recruitment of downstream homologous recombination (HR) and non-homologous end joining (NHEJ) assembly.^32^ KAT8 and ATM form a positive feedback loop. KAT8 directly interacts with the leucine zipper domain of ATM, promoting ATM autophosphorylation (Ser1981) and activation. Activated ATM in turn phosphorylates KAT8 at Thr392, regulating DDR pathway choice.^40^^,^^87^^,^^100^ In tumors, KAT8 acetylates Williams syndrome transcription factor (WSTF) at K426 to upregulate *BRCA1* and *RAD51*, enhancing HR repair capacity and cisplatin resistance.^73^ KAT8 depletion impairs recruitment of HR proteins (meiotic recombination 11 (Mre11), replication protein A 70 (RPA70), RAD51), and reduces DNA end resection efficiency, partially mediated by KAT8-proliferating cell nuclear antigen (PCNA) interaction. KAT8 promotes PCNA ubiquitination and accelerates its recruitment to damage sites, coordinating replication and repair under replication stress.^101^ In S/G2 phase, this phosphorylation event triggers dissociation of the KAT8-53BP1 complex from damage sites, relieving 53BP1-mediated inhibition of end resection and facilitating BRCA1-dependent HR. In G1 phase, this phosphorylation is diminished, enabling stable 53BP1 retention and directing repair toward NHEJ.^40^^,^^87^^,^^100^ Additionally, KAT8 interacts with DNA-dependent protein kinase catalytic subunit (DNA-PKcs) to modulate its phosphorylation, influencing NHEJ efficiency (Figure 4E).^99^

### Regulatory roles of KAT8 in autophagy

**Figure 5:**
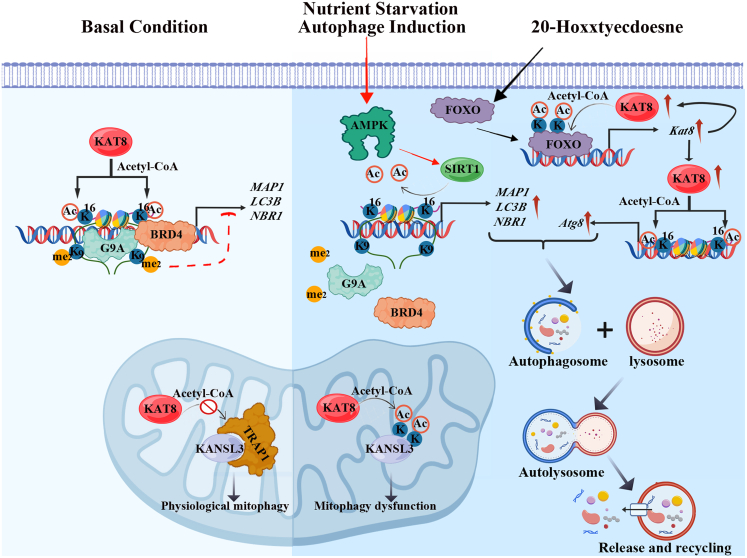
KAT8-mediated autophagy regulation Under nutrient-replete conditions, KAT8 acetylates H4K16 at the promoter regions of autophagy-regulating genes, serving as a binding platform for the reader protein BRD4. BRD4 binds and recruits the histone methyltransferase G9a, which further mediates methyl modification (H3K9me2) to repress gene expression. In mitochondria, TRAP1 interacts with KANSL3, inhibiting KAT8-mediated acetylation of KANSL3 and maintaining normal levels of mitophagy. Under nutrient deprivation or autophagic stimulation, SIRT1 deacetylates H4K16, leading to the dissociation of BRD4 and G9a. The removal of histone methyl modifications at the promoters of autophagy-regulating genes induces autophagy gene expression and triggers cellular autophagy. In diabetic models, increased mitochondrial KAT8 expression enhances KANSL3 acetylation, disrupting mitophagic balance and resulting in mitochondrial damage and dysfunction. During insect metamorphosis from larva to pupa, 20-hydroxyecdysone (20E) induces the upregulation of the transcription factor FOXO. KAT8 is not only transcriptionally activated by FOXO but also feedback-acetylates FOXO at lysines 180 and 183 to enhance its activity. Subsequently, KAT8 is recruited to the promoter region of the core autophagy gene *Atg8*, catalyzing local H4K16ac to directly promote Atg8 transcription, forming a positive feedback loop that drives the autophagic process.

KAT8 controls autophagy through a precise multi-layered dynamic network relying on site-specific acetylation of both histone and non-histone substrates (Figure 5).

Under nutrient-replete conditions, KAT8-catalyzed H4K16ac serves as a binding platform for bromodomain-containing protein 4 (BRD4). BRD4 recruits the methyltransferase G9a, which deposits the repressive mark H3K9me2. This forms a dual epigenetic repression complex that directly blocks autophagy gene transcription. Nutrient deprivation triggers SIRT1-mediated H4K16ac deacetylation, causing BRD4/G9a dissociation and relieving autophagy gene repression.^88^^,^^102^^,^^103^^,^^104^ This precise molecular timing dictates cell fate under stress. Beyond histones, KAT8 directly modifies non-histone substrates to manage organelle clearance. In diabetic cardiomyopathy, the chaperone TNF receptor-associated protein 1 (TRAP1) inhibits the acetylation of KANSL3. Pathological KANSL3 acetylation disrupts this balance, leading to mitophagy dysfunction and mitochondrial damage.^105^ KAT8 also forms specific hormone-driven feedback loops to amplify autophagy. In insect metamorphosis, 20-hydroxyecdysone induces forkhead box O (FOXO), which recruits KAT8 to the *Atg8* promoter, forming a positive feedback loop that drives autophagy.^10^ In infection models, KAT8 acetylates nuclear factor erythroid 2-related factor 2 (NRF2) to promote its nuclear retention and drive heme oxygenase 1 (HO-1) expression. This links autophagy regulation directly to ferroptosis.^66^ In summary, KAT8 serves as a central node that integrates metabolic and hormonal signals into a unified mechanistic framework to govern autophagy. The disruption of these precisely tuned homeostatic mechanisms is a central theme underlying KAT8’s involvement in a wide spectrum of human diseases, which we explore in the following section.

### KAT8 in human diseases: A context-dependent regulator of cell fate

The functional pleiotropy of KAT8 is nowhere more evident than in human diseases, where it exhibits remarkable context dependency. KAT8 acts as a central node in a regulatory network, where its ultimate impact on cell fate is dictated by cell type-specific interactions, distinct complex partners (MSL vs. NSL), and the upstream signaling pathways. This section synthesizes findings across oncology, neurobiology, immunology, and metabolic research into a unified framework. A crucial caveat, highlighted by recent work in non-transformed cells,^23^ is that H4K16ac does not invariably activate transcription; in normal mammary epithelial cells, it primarily regulates DNA replication timing. However, in the context of malignancy—where KAT8 is frequently overexpressed, metabolic cofactors (e.g., acetyl-CoA, lactate) are abundant, and specific chromatin targeting is rewired—the same enzymatic activity becomes a driver of oncogenic transcription, metabolic reprogramming, and immune evasion. This analysis establishes that KAT8’s function is not fixed but is dynamically tuned by the local molecular environment, providing a conceptual foundation for understanding its “double-edged sword” nature across the spectrum of human pathologies.

### KAT8 in malignancy: A unified framework for context-dependent functions

The involvement of KAT8 in cancer is characterized by profound context dependency. Rather than functioning as a universal oncogene or a simple tumor suppressor, KAT8 acts as a versatile epigenetic hub whose output is dictated by tissue lineage, complex assembly, substrate availability, and microenvironmental cues. We have summarized the research evidence for KAT8 across more than a dozen cancer types in Table 2. By integrating KAT8’s roles, we construct a unified mechanistic framework that organizes its oncogenic and tumor-suppressive activities into discrete functional modules.

**Table 2:** The expression of KAT8 in different types of cancer

Type of cancer	Method of detection	Differential expression	Prognosis	Mechanisms impacting tumorigenesis	Reference
**Upregulated expression**					

NSCLC	WB, qPCR, etc.	overexpression/high expression	associated with malignant progression	acetylates SIRT6, relieving ZEB2 suppression, promoting invasion/metastasis. acetylates BRM, inactivating it and releasing growth suppression; acetylates NRF2, enhancing nuclear retention and stress adaptation; acetylates WSTF, enhancing DNA repair and cisplatin resistance; enhances H4K16ac at *SKP2* promoter, upregulating SKP2 to promote S-phase progression	Wang et al.^14^; Singh et al.^40^; Sui et al.^73^; Zhao et al.^78^; Kahali et al.^79^; Chen et al.^106^
ESCC	HC, WB	upregulated	poor prognosis (advanced stage, lymph node metastasis)	stabilized by USP10-mediated deubiquitination, leading to nuclear accumulation; recruited by c-Jun to ANXA2 promoter; H4K16ac activates Wnt/β-catenin pathway; directly acetylates c-Myc (K445), enhancing stability and sustaining proliferation	Zhang et al.^12^; Li et al.^13^
Glioblastoma (GBM)	gene expression, IHC	high expression	controversial	core of NSL complex; affects H4K16ac, chromatin structure, and gene expression; loss causes genomic instability; downregulation reduces H4K16ac, silencing tumor suppressors (e.g., TMS1); knockdown reduces proliferation, causes G2/M arrest, decreases EGFR pathway activity; inhibits apoptosis, maintaining cell survival	Wang et al.^14^; Dong et al.^107^; Yin et al.^108^; Liu et al.^109^; Ferreyra Solari et al.^110^
Bladder cancer	IHC	upregulated	poor overall survival	acetylates YEATS4, stabilizing it, promoting DNA repair, genomic stability, and proliferation.	Xie et al.^56^
Cervical cancer	IHC	upregulated	shorter relapse-free survival	enhances HPV18 E7 transcription and acetylates E7 protein; promotes pRb/E2F1 interaction, driving proliferation; its activity regulated by SIRT1-mediated deacetylation	Lu et al.^81^; Xu et al.^89^
Oral tongue squamous cell carcinoma	IHC, PCR, etc.	upregulated	poor overall and disease-free survival	promotes proliferation, clonogenicity, and tumorigenesis by targeting EZH2 (enhancing its expression)	Wang et al.^14^; Li et al.^111^

**Bidirectional/complex patterns**					

Colorectal cancer (CRC)	mRNA detection	downregulated (in >57% patients)	low expression correlates with lymph node metastasis and advanced stage	acetylation regulated by GCN5/SIRT6; decreased acetylation inhibits HSL transcription, suppressing migration/invasion; catalyzes eEF1A2 lactylation, activating protein synthesis to drive proliferation	Xie et al.^83^; Qiu et al.^112^; Cao et al.^113^
Hepatocellular Carcinoma (HCC)	WB, IHC	bidirectional (both low and high reported)	complex, mechanism-dependent	low expression: decreases H4K16ac at OAT2 promoter, inducing chemo-resistance; inhibitory role: acetylation of FASN or ERα inhibits fatty acid synthesis or exerts tumor-suppressive effects; high expression: (a) increases H4K16ac, activating pro-invasion genes (e.g., AXL), (b) increases H3K9ac at DDX41 promoter, upregulating ribosome biogenesis; (c) acetylates KDM1A, inducing anti-apoptotic signaling and sorafenib resistance, (d) acetylates PCK2, stabilizing it and promoting metabolic reprogramming/resistance; interacts with notch signaling; regulates transcription factors affecting HBV replication	Li et al.^65^; Lin et al.^68^; Liu et al.^70^; Wang et al.^74^; Wei et al.^90^; Lv et al.^114^; Jing et al.^115^; Wang et al.^116^; Poté et al.^117^
Breast cancer	expression analysis, WB, IHC	downregulated in BCSCs; active in ER + cancer	associated with stem cell maintenance, therapy tolerance, progression	acetylates WSTF, enhancing DNA repair and EMT gene expression; maintains H4K16ac at TMS1 promoter, preventing its silencing; via MSL complex, acetylates AURKB, stabilizing it and promoting proliferation; in ER + cancer, recruited by PHF20/G9a to Erα targets, catalyzing H4K16ac to activate transcription	Kapoor-Vazirani et al.^52^; Miao et al.^64^; Zhang et al.^72^; Zekri et al.^118^; Liu et al.^119^
Renal cell carcinoma (RCC)	gene expression, IHC	complex: mRNA up in KIRC; protein down in 90.5% of patients	high mRNA in KIRC correlates with poor prognosis; protein downregulation linked to tumorigenesis	upregulates Skp2, promoting S-phase progression; lactylates MDH2 (K239), enhancing activity, promoting ATP/NADPH production, strengthening metabolism and antioxidant capacity; reduced protein linked to decreased H4K16ac and renal carcinogenesis	Singh et al.^40^; Tang et al.^85^; Cao et al.^113^; Wang et al.^120^
Endometrial cancer	IHC, WB	complex: MOF downregulated but correlates with ERα; KAT8 upregulated in endometrioid adenocarcinoma	MOF low correlates with high grade (poor prognosis); MOF high correlates with ERα high (better survival); KAT8 high is an independent risk factor (poor prognosis)	KAT8 as suppressor: acetylates ERα, maintaining its stability and regulating target genes (e.g., DRAM1, TAGLN) to inhibit growth; KAT8 as promoter: estrogen upregulates KAT8 via PI3K/Akt and Ras pathways; high KAT8 promotes proliferation, migration, invasion, inhibits apoptosis, enriches cell cycle/spliceosome pathways.	Wang et al.^14^; Wu et al.^121^; Qi et al.^122^
Acute myeloid leukemia (AML)	RNA analysis, WB	high in MLL-AF9 leukemia; generally downregulated in AML patients, rises during differentiation	MOF deletion prolongs mouse survival; KAT8 downregulation correlates with poor prognosis; restored expression may promote differentiation	HAT activity crucial for leukemia cell growth; deletion disrupts genomic stability; binds and inhibits MN1 promoter, regulating NF-κB and inflammatory cytokines (e.g., IL6, CXCL8), affecting differentiation	Valerio et al.^15^; Zhao et al.^123^
Gallbladder carcinoma	IHC	expression not explicitly stated (mutations exist)	high KAT8 correlates with poor overall and disease-free survival	promotes proliferation, clonogenicity, and tumorigenesis by targeting EZH2 (enhancing its expression)	Pandey et al.^124^

### Core oncogenic mechanisms shared across cancer types

#### Transcriptional reprogramming through chromatin remodeling

KAT8 drives oncogenic transcriptional programs by catalyzing H4K16ac at target gene promoters and enhancers, often with lineage-specific transcription factors. This mechanism is conserved across multiple malignancies but yields distinct target gene repertoires depending on cellular context. In ESCC, ubiquitin-specific peptidase 10 (USP10) stabilizes KAT8 by removing ubiquitin from K410. The stabilized KAT8 is then recruited by the transcription factor c-Jun to the *ANXA2* promoter. There, H4K16ac activates *ANXA2* transcription and triggers Wnt/β-catenin signaling, which drives proliferation and metastasis.^13^ Additionally, KAT8-mediated H3K9ac at the *DEAD-box helicase 41* (*DDX41)* promoter enhances ribosome biogenesis and supports rapid proliferation in hepatocellular carcinoma (HCC).^114^ In glioblastoma (GBM), KAT8 overexpression correlates with aberrant activation of the epidermal growth factor receptor (EGFR) pathway, while KAT8 knockdown induces G2/M arrest and reduces EGFR activity.^107^ KAT8 overexpression also enhances NSCLC migration and adhesion.^40^ In cell cycle regulation, KAT8 elevates H4K16ac at the promoter of *SKP2*, upregulating SKP2 expression and accelerating S-phase progression.^14^^,^^40^

#### Non-histone acetylation that rewires oncogenic signaling

KAT8 directly acetylates non-histone substrates, modulating their stability, subcellular localization, and interaction networks. This layer of regulation allows KAT8 to rapidly reprogram core signaling pathways. Substrate stabilization—in ESCC, KAT8 acetylates c-Myc at K445, preventing ubiquitin-proteasome degradation, leading to c-Myc accumulation and expression of downstream targets (e.g., *NPM1*, *PPAT*). The selective KAT8 inhibitor CHI KAT8i5 blocks this axis and suppresses ESCC tumor growth,^12^ DNA repair, and chemoresistance. In NSCLC, KAT8 acetylates WSTF at K426, upregulating *BRCA1* and *RAD51* expression, thereby potentiating HR repair and conferring cisplatin resistance.^73^ In bladder cancer, KAT8-mediated acetylation of YEATS domain-containing 4 (YEATS4) stabilizes the protein and promotes DDR; KAT8 inhibition sensitizes bladder cancer cells to cisplatin.^56^ Epithelial-mesenchymal transition (EMT) and metastasis. In NSCLC, KAT8 acetylates SIRT6 at K128/K160/K267. These acetylations inhibit SIRT6 deacetylase activity, relieving repression of *zinc finger e-box-binding homeobox 2 (ZEB2)*. The ensuing ZEB2 upregulation activates EMT programs, promoting invasion and metastasis.^78^

#### Metabolic reprogramming and adaptation to oxidative stress

KAT8 acts as a metabolic sensor and effector through acetylation, lactylation, and other acyl modifications. This function is particularly relevant in the tumor microenvironment, where altered metabolite levels (lactate, acetyl CoA) directly influence KAT8 activity, and KAT8 in turn reprograms cancer cell metabolism. In kidney renal clear cell carcinoma (KIRC), KAT8 catalyzes malate dehydrogenase 2 (MDH2) lactylation at K239. This modification enhances MDH2 enzymatic activity, increasing the reduced nicotinamide adenine dinucleotide (NADH)/nicotinamide adenine dinucleotide (NAD^+^) ratio and nicotinamide adenine dinucleotide phosphate (NADPH) production, which strengthens mitochondrial respiration and antioxidant capacity, thereby driving tumor progression.^85^ In CRC, a high lactate microenvironment induces KAT8-dependent eukaryotic translation elongation factor 1 alpha 2 (eEF1A2) lactylation at K408, activating tumor-specific protein synthesis and promoting malignant proliferation. The KAT8 inhibitor MG149 abrogates this lactylation event and suppresses CRC cell growth.^83^ In HCC, KAT8 exerts dual and opposing effects. Acetylation of phosphoenolpyruvate carboxykinase 2 (PCK2) at K491 stabilizes the protein, promotes mitochondrial gluconeogenesis, and contributes to sorafenib resistance. Conversely, KAT8-mediated acetylation of fatty acid synthase (FASN) targets it for tripartite motif-containing 21 (TRIM21)-dependent ubiquitination and degradation, thereby inhibiting *de novo* lipogenesis and suppressing tumor growth.^74^^,^^90^ This “one enzyme, two directions” paradigm illustrates contextual tuning of KAT8 function in metabolic networks.

#### Immune checkpoint regulation and tumor immune evasion

KAT8 contributes to immune evasion through two synergistic mechanisms: direct chromatin activation of the *PD-L1* promoter and acetylation-driven activation of the transcription factor IRF1. In multiple solid tumor types, the MSL complex catalyzes H4K16ac at the *PD-L1* promoter, enhancing PD-L1 expression.^125^ More importantly, interferon gamma (IFNγ)-induced IRF1 recruits KAT8 through liquid-liquid phase separation. KAT8 then acetylates IRF1 at K78, which enhances the DNA-binding affinity of IRF1. Concurrently, local H4K16ac relaxes chromatin structure. These two events synergistically drive *PD-L1* transcription and suppress CD8^+^ T cell-mediated antitumor immunity. The blocking peptide (2142-R8) that disrupts this phase separation reduces PD-L1 and enhances antitumor immunity.^55^ In pancreatic cancer, KAT8 acetylates selenoprotein P1 (SEPP1) at K247/K249, increasing its stability and secretion. Secreted SEPP1 binds to the lipoprotein receptor-related protein 8 (LRP8) on myeloid-derived suppressor cells (MDSCs), triggering MDSC apoptosis and relieving immunosuppression to activate CD8^+^ T cell responses.^75^ This finding reveals an unexpected, context-dependent immunostimulatory role of KAT8 within the tumor microenvironment.

It is worth noting that the transcriptional role of H4K16ac is context-dependent. In non-transformed human mammary epithelial cells, global H4K16ac loss does not significantly alter gene expression. This apparent discrepancy can be reconciled by recognizing that in cancer cells—where KAT8 is often overexpressed and the MSL complex is aberrantly recruited to specific oncogene promoters (e.g., *SKP2, PD-L1*)—H4K16ac drives localized transcriptional activation. Thus, the oncogenic functions of KAT8 reflect pathological hijacking of a modification that, under normal homeostatic conditions, primarily serves to maintain replication timing fidelity.

#### Tumor-suppressive functions of KAT8: Mechanisms and contexts

In a subset of malignancies and under specific genetic or microenvironmental conditions, KAT8 acts as a tumor suppressor. Understanding the molecular basis of this “double-edged sword” is imperative for designing precision therapeutic strategies.

#### Maintenance of tumor suppressor expression and genomic stability

In breast cancer, KAT8 maintains H4K16ac at the promoter of the tumor suppressor gene *TMS1/ASC*, preventing epigenetic silencing of TMS1/ASC. Loss of KAT8 correlates with TMS1/ASC silencing, aggressive disease, and chemoresistance.^52^ In epithelial ovarian cancer, high KAT8 expression is paradoxically associated with favorable prognosis. Mechanistically, KAT8 (via the MSL1v1 complex) acetylates lysine-specific histone demethylase 1 (LSD1) at K432/K433/K436. This acetylation weakens LSD1’s nucleosome binding and inhibits its H3K4 demethylase activity, thereby preserving an epithelial phenotype and suppressing EMT and metastasis.^69^

#### Antagonism of pro-tumorigenic metabolic programs

In HCC, KAT8-mediated acetylation of FASN promotes its interaction with TRIM21, leading to FASN degradation via the ubiquitin-proteasome pathway. This reduces *de novo* lipogenesis and suppresses tumor growth. Histone deacetylase 3 (HDAC3) counteracts this acetylation and stabilizes FASN, revealing a dynamic acetylation-deacetylation balance that controls lipid metabolism.^74^ KAT8 modulates CRC progression via non-histone modifications: its autoacetylation (K168/K175) is dynamically regulated by general control of amino acid synthesis 5 (GCN5) and SIRT6. When acetylated, KAT8 exhibits reduced binding to the hormone-sensitive lipase (*HSL*) promoter, repressing *HSL* transcription and inhibiting CRC cell migration and invasion.^112^ In endometrial cancer, KAT8 acetylates estrogen receptor α (ERα) at K266/K268/K299, enhancing ERα stability and upregulating tumor suppressor genes such as *DRAM1* and *TAGLN*. However, estrogen-driven phosphatidylinositol 3-kinase/AKT serine-threonine kinase (PI3K/Akt) signaling can also upregulate KAT8 expression, and at very high levels KAT8 switches to promoting proliferation, migration, and survival, illustrating a dose-dependent functional switch.^121^^,^^122^

#### Suppression of metastatic cytoskeletal remodeling

In ESCC, while KAT8 acts as an oncogene in early stage tumors, its expression is significantly reduced in highly metastatic cells. KAT8 directly acetylates fascin 1 (FSCN1) at K41, inhibiting its F-actin bundling activity, thereby reducing filopodia and invadopodia formation and suppressing cancer cell migration and invasion. Low KAT8 expression and reduced FSCN1 acetylation correlate with lymph node metastasis and poor prognosis in ESCC patients.^60^

The seemingly contradictory roles of KAT8 can be reconciled by recognizing that its functional output is not an intrinsic property but is collectively dictated by five interacting determinants: the identity of the assembled complex, the preference for specific substrates, the nature of microenvironmental signals, the precise level of KAT8 expression, and the cell lineage context. First, complex assembly serves as a primary switch. When KAT8 is incorporated into the MSL complex, it predominantly engages oncogenic axes such as PD-L1 activation and c-Myc stabilization.^72^^,^^125^ In contrast, assembly into the KAT8-MSL1v1 complex directs KAT8 toward tumor-suppressive substrates including p53 and LSD1.^53^ Second, substrate preference follows from complex assembly. In its oncogenic mode, KAT8 targets metabolic enzymes like MDH2 and PCK2.^12^ Third, microenvironmental signals gate KAT8 activity. High lactate, IFNγ, and transforming growth factor β (TGF-β) tend to promote its oncogenic functions,^55^^,^^83^^,^^126^ whereas nutrient deprivation and DNA damage signals favor tumor-suppressive outcomes.^40^^,^^103^ Fourth, the expression level of KAT8 follows a biphasic pattern. Moderate overexpression, as seen in NSCLC and ESCC, drives proliferation programs. However, very low expression leads to genomic instability, and paradoxically excessive expression may cause metabolic exhaustion, both of which can suppress tumor growth.^38^^,^^74^^,^^113^ Fifth, cell lineage dictates how KAT8 rewires the transcriptional landscape. In NSCLC, ESCC, GBM, and bladder cancer, KAT8 acts predominantly as an oncoprotein. In ovarian cancer, a subset of HCC and CRC, and endoplasmic reticulum (ER)-positive endometrial cancer, it more frequently exerts tumor-suppressive effects.^69^^,^^108^^,^^113^^,^^121^^,^^124^

For KAT8-driven malignancies where the oncogenic modules predominate, the development of highly selective KAT8 inhibitors is warranted. Preclinical candidates such as CHI-KAT8i5 and compound 34 have shown promise in ESCC and NSCLC models.^12^^,^^127^ Conversely, where KAT8 functions as a tumor suppressor, systemic inhibition should be avoided. In these contexts, strategies to restore or enhance KAT8 activity, such as inhibiting its negative regulators (SIRT1-mediated deacetylation or mouse double minute 2 homolog-mediated ubiquitination), may offer therapeutic benefit.^30^^,^^89^^,^^115^ Furthermore, the expression level of KAT8 and its post-translational modifications, particularly autoacetylation at K274 and phosphorylation at T392, hold potential as predictive biomarkers to guide patient stratification for KAT8-targeted therapies.^31^^,^^40^ The same context-dependent principles govern KAT8’s function in normal cellular metabolism and related diseases, as detailed in the following section.

### KAT8 as a central regulator of cellular metabolism: Common principles across tissues and diseases

**Figure 6:**
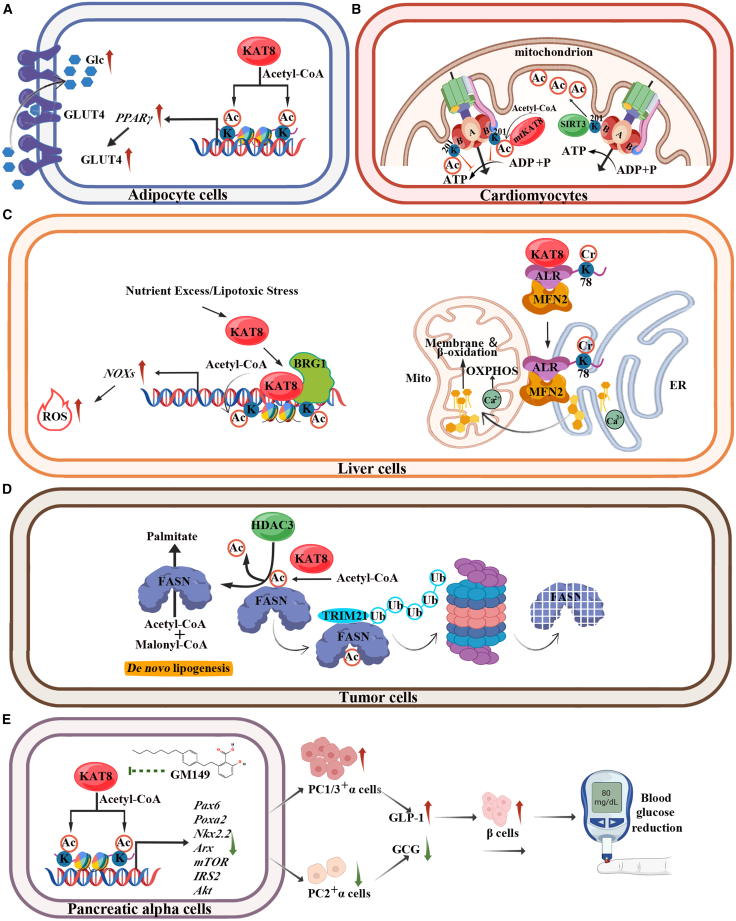
KAT8 regulates cellular metabolism in a cell type-specific manner (A) In adipocytes, KAT8 acetylates H4K16 at the *PPARγ* promoter to enhance the transcription of PPARγ and its downstream target GLUT4, thereby promoting glucose uptake. (B) In cardiomyocytes, mitochondrial KAT8 acetylates ATP5B at lysine 201, which inhibits mitochondrial ATP synthesis and contributes to mitochondrial dysfunction and heart failure. (C) Under nutrient excess or lipotoxic stress in liver cells, BRG1 recruits KAT8 to the promoters of NADPH oxidase genes (e.g., *NOX1*). KAT8-mediated H4K16 acetylation upregulates NOX expression and increases ROS production, promoting oxidative stress, inflammation, and fibrosis in NASH. In hepatocellular carcinoma, KAT8-mediated crotonylation of ALR at K78 promotes ALR-MFN2 interaction and stabilizes mitochondria-ER contact sites (MAMs), facilitating lipid transfer and Ca2^+^ exchange to support mitochondrial membrane formation and energy metabolism. (D) In tumor cells, KAT8 acetylates FASN, leading to TRIM21-dependent ubiquitination and degradation of FASN and thereby suppressing *de novo* lipogenesis. HDAC3 counteracts this acetylation and stabilizes FASN. (E and F) In pancreatic α-cells, inhibition or knockout of KAT8 reduces H4K16ac levels and downregulates a set of genes (e.g., *Pax6*, *Foxa2*, *Nkx2.2*, *Arx*, *mTOR*, *IRS2*, *Akt*). This results in an increase in PC1/3^+^ α-cells and GLP-1 secretion, a decrease in PC2^+^ α-cells and glucagon secretion, and promotion of β-cell proliferation and insulin release, collectively leading to blood glucose reduction and improved insulin sensitivity in diabetic models.

KAT8 functions as a master rheostat of cellular metabolism. It integrates nutrient and energy signals to orchestrate transcriptional and post-translational programs. The same enzymatic activities drive histone acetylation and non-histone acylation in cancer. These activities also govern metabolic adaptation in adipose tissue, liver, heart, and pancreatic islets. As in oncology, the metabolic output of KAT8 is highly context-dependent: it can promote glucose uptake in adipocytes while suppressing ATP synthesis in cardiomyocytes, and either enhance or inhibit lipogenesis depending on the cellular milieu. The key pathways discussed are illustrated in Figure 6.

#### KAT8 as a transcriptional gatekeeper of glucose and energy metabolism

KAT8 controls systemic glucose homeostasis through epigenetic regulation. In adipose tissue (Figure 6A), KAT8-mediated H4K16ac at the *peroxisome proliferator-activated receptor gamma* (*Pparγ)* promoter enhances PPARγ transcriptional activity. This leads to upregulation of glucose transporter type 4 (GLUT4), promoting insulin-stimulated glucose uptake. Adipose-specific *Kat8* insufficiency reduces H4K16ac, represses *Pparγ* transcription, impairs glucose assimilation, and defective insulin secretion, recapitulating prediabetic features. Paradoxically, these mice are resistant to diet-induced obesity due to compromised fat storage.^128^ This finding illustrates a core principle: KAT8-mediated H4K16ac can simultaneously promote glucose disposal and limit lipid accumulation, a context-dependent duality that mirrors its “double-edged sword” behavior in cancer. In pancreatic α-cells (Figure 6E), KAT8 fine-tunes glucagon secretion and α-cell identity. *Kat8* depletion alters the proportion of PC1/3-positive versus PC2-positive α-cell subsets, leading to aberrant glucagon secretion and hypoglycemia. MOF-catalyzed H4K16ac at the promoters of key transcription factors (e.g., *paired box 6* [*Pax6*], *forkhead box A2* [*Foxa2*]) creates an open chromatin environment that facilitates transcription factor binding and transcriptional activation to maintain α-cell differentiation and function.^129^^,^^130^ Pharmacological inhibition of KAT8 with MG149 ameliorates glucose intolerance and islet dysfunction in type 2 diabetes models, suggesting that KAT8 activity in α-cells is a potential therapeutic target.^130^

#### KAT8 in mitochondrial function and oxidative phosphorylation

KAT8 controls mitochondrial energy production via a mitochondrial pool and acetylation of nuclear encoded mitochondrial proteins. In cardiomyocytes (Figure 6B), mtKAT8 (as part of the NSL complex) binds mtDNA and regulates oxidative phosphorylation. In heart failure, mtKAT8 hyperacetylates ATP synthase subunit beta (ATP5B) at K201. This acetylation directly inhibits ATP synthase activity, reducing ATP synthesis efficiency and inducing mitochondrial dysfunction, which drives cardiac remodeling. Sirtuin 3 (SIRT3) antagonizes this acetylation.^71^ In the liver (Figure 6C), KAT8-mediated crotonylation of augmenter of liver regeneration (ALR) at K78 strengthens ALR-mitofusin 2 (MFN2) interaction, stabilizing mitochondria-ER contact sites (MAMs). This stabilization preserves lipid transport and oxidative phosphorylation. Loss of this modification disrupts MAM integrity, promotes intracellular lipid accumulation, and contributes to metabolic dysfunction-associated fatty liver disease (MASLD).^19^

#### KAT8-mediated lipid metabolism: Opposing effects in health and disease

KAT8 regulates lipid homeostasis through histone acetylation-dependent transcription and direct acetylation of lipogenic enzymes. In non-alcoholic steatohepatitis (NASH), KAT8 is recruited by the chromatin remodeler Brahma-related gene 1 (BRG1) to *NOX* gene promoter. KAT8-catalyzed H4K16ac activates *NOX* transcription, elevating reactive oxygen species (ROS) and promoting oxidative stress, inflammation, and fibrosis, all hallmarks of NASH progression.^131^ In contrast, in cancer cells, KAT8 directly acetylates FASN, targeting it for TRIM21-dependent degradation. This acetylation-dependent degradation inhibits *de novo* fatty acid synthesis and suppresses tumor growth^74^ (Figure 6D). These opposing outcomes underscore a recurring theme: the same enzyme, acting on different substrates or in different subcellular compartments, can exert diametrically opposite effects on lipid metabolism.

#### Tissue-specific and pathophysiological integration

The metabolic functions of KAT8 are highly tissue-specific. In the pancreas, MOF maintains α-cell identity and glucagon secretion.^129^ In the heart, excessive mtKAT8 activity contributes to energetic failure.^71^ KAT8 acts as a metabolic hub that integrates organ-specific transcriptional networks with post-translational control of metabolic enzymes. The direction of its metabolic output (protective versus pathological) is determined by the local balance of cofactors, the availability of distinct complex partners, and the underlying disease context.

### KAT8 in neurological disorders

**Figure 7:**
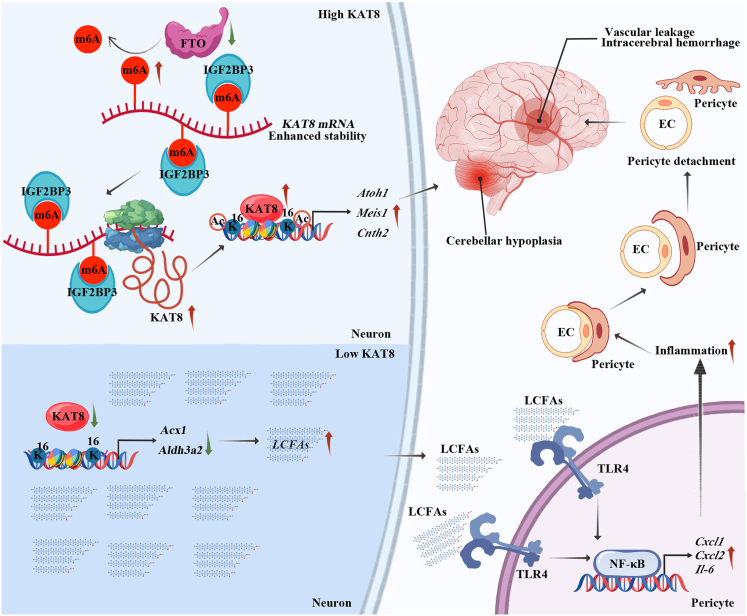
KAT8 regulates neurodevelopment and brain homeostasis through RNA epigenetic control, and its dysregulation leads to cerebrovascular and inflammatory pathologies (Left) m6A-mediated regulation of KAT8 expression: FTO deletion increases m6A methylation on Kat8 mRNA, which is recognized by the reader protein IGF2BP3 to enhance mRNA stability and elevate KAT8 expression. This establishes an RNA-to-histone modification axis that drives excessive H4K16ac and chromatin over-opening, leading to dysregulated expression of neurodevelopmental genes and cerebellar hypoplasia. Conversely, low KAT8 expression in neurons disrupts metabolic homeostasis, contributing to downstream inflammatory responses. (Right) Pathological consequences of KAT8 dysregulation: high KAT8 in the neurovascular unit promotes pericyte detachment from endothelial cells (ECs) through upregulation of genes such as Atoh1, Meis1, and Cntb2, resulting in vascular leakage and intracerebral hemorrhage. Low KAT8 in neurons leads to the accumulation of long-chain fatty acids (LCFAs), which activate the TLR4-NF-κB pathway in pericytes. This triggers the expression of pro-inflammatory cytokines (e.g., *Cxcl1*, *Cxcl2*, *Il-6*), promoting inflammation and compromising blood-brain barrier integrity.

KAT8 governs nervous system homeostasis through H4K16ac at neural loci, NSL complex-mediated mitophagy, and MSL complex-mediated transcriptional precision. Disruption of these modules leads to neurodevelopmental and neurodegenerative disorders (Figure 7).

#### RNA epigenetic control of KAT8 expression in cerebellar development

KAT8 expression is finely tuned by RNA N6 methyladenosine (m6A) methylation. Fat mass and obesity-associated protein (FTO) deletion elevates m6A methylation of *Kat8* mRNA, which is recognized by the reader protein insulin-like growth factor 2 mRNA binding protein 3 (IGF2BP3). This recognition enhances Kat8 mRNA stability and increases KAT8 protein levels. Elevated KAT8 then promotes excessive H4K16ac and aberrant chromatin relaxation at neural developmental gene loci, leading to dysregulated gene expression and cerebellar hypoplasia. The KAT8 inhibitor MC4171 partially rescues this phenotype, demonstrating a direct link from RNA epigenetics to histone modification in neurodevelopment.^132^

#### Metabolic and vascular consequences of KAT8 dysregulation in the brain

KAT8 maintains metabolic homeostasis in neural cells, and its dysregulation leads to secondary cerebrovascular and inflammatory pathologies. Neural-specific *Kat8* deletion downregulates metabolic genes, especially those involved in fatty acid β-oxidation, causing accumulation of free long chain fatty acids. These fatty acids activate the toll-like receptor 4 (TLR4) nuclear factor kappa B (NF-κB) inflammatory pathways in cerebrovascular pericytes, disrupting blood-brain barrier integrity and leading to intracerebral hemorrhage.^133^ Conversely, when KAT8 expression is elevated in the neurovascular unit, it promotes pericyte detachment from endothelial cells through upregulation of genes such as *Atoh1*, *Meis1*, and *Cntb2*, resulting in vascular leakage.^133^ These findings establish that proper KAT8 dosage is requisite for both neuronal function and cerebrovascular integrity. Figure 7 illustrates the neurodevelopmental and vascular consequences of KAT8 dysregulation.

#### KAT8 in neurodegenerative diseases: Mitophagy and beyond

KAT8 and KANSL1 are required for PTEN-induced kinase 1(PINK1) stability and Parkin-mediated mitophagy. KANSL1, as a scaffold component of the NSL complex, regulates KAT8 function and substrate targeting within the complex, influencing H4K16 acetylation and downstream gene expression, rather than directly regulating KAT8 expression levels. Knockdown of either impairs damaged mitochondrial clearance in Parkinson’s disease (PD) models.^134^ MG149 reduces PINK1 activity and mitophagy markers.^135^ The *KAT8* genomic locus is a shared risk locus for Alzheimer’s disease (AD) and PD, and is integrated into the autophagy lysosomal dysfunction pathway.^136^^,^^137^ KAT8 expression is regulated by KANSL1 and correlates with AD risk in apolipoprotein E ε4 (APOE ε4) negative individuals, suggesting its role in complex genetic backgrounds.^138^ Analysis of the co-expression network of NSL complex-related genes in the human brain shows enrichment in prefrontal cortical neurons, with tight associations with PD-L1-related pathways including autophagy and chromatin organization.^139^ In addition, a functional frameshift mutation in KAT8 identified in individuals with 22q11.2 deletion syndrome suggests its involvement in schizophrenia pathogenesis by perturbing brain development, supporting a polygenic cumulative model for this disorder.^140^

Beyond the catalytic role of KAT8, pathogenic variants in KAT8 partner subunits reveal disease-specific vulnerabilities. These findings establish “MSL/NSL complex” as a distinct class of neurodevelopmental disorders where the specific subunit affected dictates the molecular and clinical phenotype. In X-linked MSL3- related disorder (MRXSBA), patient fibroblasts show global H4K16ac reduction despite normal KAT8 levels, caused by MRG domain mutations that destabilize the MSL complex, leading to mis-regulation of Notch, Hox, and Wnt pathways.^141^^,^^142^ In contrast, *de novo* MSL2 variants cause a distinct neurodevelopmental syndrome MSL2-associated neurodevelopmental syndrome (MANDS) without global H4K16ac loss but with dysregulation of specific targets such as ZNF185 and BEX2.^143^ NSL subunit KANSL1 haploinsufficiency causes Koolen-de Vries syndrome^92^^,^^134^; KANSL1 also regulates PINK1-dependent mitophagy^134^ and is a risk gene for progressive supranuclear palsy.^137^ Thus, the affected subunit dictates the molecular pathology: global epigenetic collapse (MSL3), targeted transcriptional dysregulation (MSL2), or impaired organelle quality control (KANSL1).

### Mechanistic links between KAT8, immune inflammation, aging, and other diseases

KAT8 exhibits striking functional duality in the immune system, inflammatory disorders, and aging processes. This contextual output is dictated by multiple interacting determinants: the nature of the upstream stimulus (pathogen-associated vs. damage-associated signals), the availability of metabolic cofactors (lactate, acetyl CoA), and the cell type or tissue microenvironment.

#### Trained immunity versus antiviral negative feedback: A metabolic epigenetic switch

KAT8 acts as a metabolic epigenetic rheostat that can either prime innate immunity for enhanced recall responses or restrain excessive antiviral inflammation. In invertebrate trained immunity, shrimps primed with ultraviolet inactivated white spot syndrome virus (UV WSSV) upregulate KAT8 via FOXO. The accompanying virus induced metabolic reprogramming elevates acetyl CoA levels, which serves as the co-substrate for KAT8. KAT8 then deposits H3K27ac at promoters of glycolytic genes (*Hk2*, *Pk*, *Ldh*) and the NF-κB homolog Dorsal, establishing a feed-forward epigenetic loop. Upon secondary viral challenge, the pre-positioned H3K27ac enables rapid transcriptional activation of antiviral effectors such as Vago5. This constitutes a form of innate immune memory.^58^ Notably, while KAT8-mediated H3K27ac is classically catalyzed by CBP/p300 in mammals, its emergence in shrimp likely represents an invertebrate-specific adaptation to metabolic surges during trained immunity, exploiting KAT8’s substrate promiscuity in the absence of highly redundant KAT3a/b homologs. In mammals, rather than H3K27ac, this catalytic versatility manifests primarily as novel acylation (e.g., lactylation and propionylation), highlighting how cellular metabolic states can expand KAT8’s epigenetic regulatory scope beyond its canonical H4K16ac substrate.

**Figure 8:**
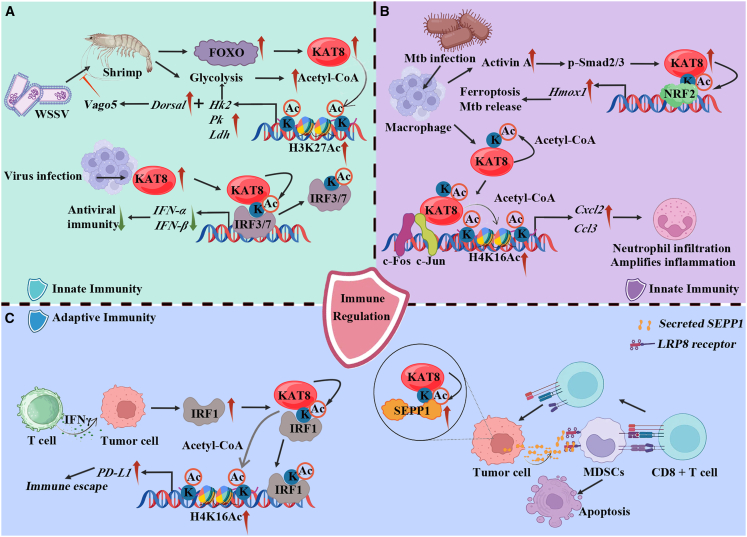
Functional roles and regulatory mechanisms of KAT8 in immunity and disease (A) Trained immunity and metabolic reprogramming: following priming with ultraviolet-inactivated white spot syndrome virus (UV-WSSV) in shrimp, FOXO-mediated upregulation of KAT8 couples with enhanced glycolytic flux and acetyl-CoA production. KAT8 deposits H3K27ac at promoters of metabolic (e.g., *Hk2*, *Pk*, *Ldh*) and immune genes, including the NF-κB homolog *Dorsal*. This establishes a feed-forward loop that potentiates the expression of effector molecules like the antiviral cytokine Vago5 upon rechallenge, constituting a trained immune state. Fine-tuning of antiviral responses: viral infection induces KAT8 expression in immune cells. KAT8 directly acetylates transcription factors IRF3 and IRF7, attenuating their DNA-binding affinity and subsequent IFN-α/β production. This negative feedback mechanism prevents excessive inflammation and immunopathology. (B) Infection and sterile inflammation: during Mtb infection, Activin A signaling via SMAD2/3 upregulates KAT8 in macrophages. KAT8 acetylates the transcription factor NRF2, stabilizing its nuclear localization and transactivation of target genes such as heme oxygenase-1 (*Hmox1*). This promotes ferroptosis, a microenvironment favorable for bacterial persistence. In sterile inflammation such as psoriasis, KAT8 autoacetylation enables its recruitment by AP-1 (c-Fos/c-Jun) to chemokine gene promoters (e.g., *Cxcl2*, *Ccl3*). Catalysis of H4K16ac facilitates their transcription, driving neutrophil recruitment and amplifying tissue inflammation. (C) Dual roles in tumor immunity: KAT8 exerts opposing effects within the tumor microenvironment: promoting immune evasion. The MSL complex, containing KAT8, catalyzes H4K16ac at the *PD-L1* promoter to enhance its expression. Furthermore, IFNγ-induced IRF1 recruits KAT8, which acetylates IRF1 at K78. This modification strengthens IRF1 binding to the *PD-L1* promoter, synergizing with local H4K16ac to amplify *PD-L1* transcription and suppress CD8^+^ T cell activity. Relieving immunosuppression: in pancreatic cancer cells, KAT8 acetylates SEPP1, increasing its stability and secretion. Secreted SEPP1 engages the LRP8 receptor on myeloid-derived suppressor cells (MDSCs), triggering MDSC apoptosis and thereby attenuating a key immunosuppressive axis.

In vertebrate antiviral immunity, KAT8 functions as a negative feedback brake. Viral infection induces KAT8 expression in immune cells, and KAT8 directly acetylates IRF3 and IRF7. This acetylation occurs within their DNA binding domains and inhibits their ability to bind interferon-stimulated response elements (ISREs), thereby dampening type 1 interferon (IFN α/β) production.^61^^,^^62^ This negative regulation prevents immunopathology that would otherwise result from uncontrolled interferon signaling. Thus, the same enzyme can either enhance or suppress antiviral immunity depending on the evolutionary context (invertebrate trained immunity vs. vertebrate precision regulation) and the specific substrates engaged (histones vs. transcription factors) (Figure 8A).

#### Sterile inflammation versus infectious host defense: Opposing outcomes of KAT8 activation

KAT8 activation produces diametrically opposite outcomes in infectious versus sterile inflammatory settings, reflecting its sensitivity to upstream signaling pathways. During Mycobacterium tuberculosis (Mtb) infection, the pathogen exploits KAT8 to establish a permissive niche. The activin A-SMAD2/3 pathway is activated in infected macrophages, leading to transcriptional upregulation of KAT8. Elevated KAT8 then acetylates NRF2 at lysine residues that enhance its nuclear retention and transcriptional activity. NRF2 target HO-1 is induced, which promotes ferroptosis. Ferroptosis releases nutrients and creates an immunoprivileged microenvironment that favors Mtb persistence^66^ (Figure 8B).

In sterile skin inflammation such as psoriasis, KAT8 drives pathological neutrophil recruitment and tissue damage. KAT8 undergoes autoacetylation, which stabilizes its active conformation. The autoacetylated KAT8 is then recruited by the AP-1 transcription complex (c-Fos/c-Jun) to the promoters of chemokine genes including *Cxcl2* and *Ccl3*. KAT8-catalyzed H4K16ac at these promoters drives chemokine transcription, leading to neutrophil infiltration, extracellular trap formation, and exacerbation of psoriatic skin lesions.^17^ Pharmacological inhibition of KAT8 using the novel inhibitor KAT8-IN-1 alleviates skin inflammation in mouse models, validating KAT8 as a therapeutic target in sterile autoinflammatory conditions.

#### Opposing roles in tumor immunity: Immunosuppression versus immunostimulation

Within the tumor microenvironment, KAT8 exerts both immunosuppressive and immunostimulatory effects, often through distinct substrate and complex preferences.

On the immunosuppressive side, KAT8 drives PD-L1 upregulation and CD8^+^ T cell exhaustion through MSL complex-catalyzed H4K16ac at the *PD-L1* promoter and IRF1 acetylation at K78 via liquid-liquid phase separation, as detailed in “trained immunity versus antiviral negative feedback: a metabolic epigenetic switch.” Conversely, KAT8 can also activate antitumor immunity in specific cancer types. In pancreatic cancer, KAT8 acetylates SEPP1 (K247/K249) to promote MDSC apoptosis and CD8^+^ T cell-mediated antitumor immunity, as detailed in “trained immunity versus antiviral negative feedback: a metabolic epigenetic switch.”^75^ Therefore, KAT8 can be either a barrier to immunotherapy or a facilitator of immune activation, depending on the cancer type and the specific substrate effector axis engaged (Figure 8C).

#### Tissue-specific fibrosis: KAT8 as a pro-fibrotic or anti-fibrotic regulator

KAT8 exhibits organ-specific and pathway-specific roles in fibrosis, further illustrating its context dependency. In skin and pulmonary fibrosis, TGF-β signaling downregulates KAT8 expression in fibroblasts via SMAD3. The reduction in KAT8 leads to decreased H4K16ac at the promoters of autophagy-related genes, relieving their transcriptional repression. This de-repression triggers aberrant autophagy activation, which in turn promotes myofibroblast transdifferentiation and excessive collagen deposition. Consistent with this mechanism, KAT8 overexpression restores H4K16ac levels, inhibits autophagic flux, and alleviates fibrosis in preclinical models.^126^

In liver fibrosis, however, KAT8 plays a direct pro-fibrotic role. KAT8 acts as a transcriptional coactivator of serum response factor (SRF). Together, SRF and KAT8 are recruited to the promoters of *neutrophil cytosolic factor 1 and 2* (*NCF1/2*), which encode subunits of the NADPH oxidase complex. KAT8-catalyzed H4K16ac at these promoters enhances NCF1/NCF2 expression, leading to increased ROS production. ROS activate hepatic stellate cells, the primary fibrogenic cells in the liver, driving fibrotic progression.^144^ Thus, KAT8 functions as an anti-fibrotic factor in skin and lung (by restraining autophagy) but as a pro-fibrotic factor in liver (by promoting ROS generation). This dichotomy underscores the importance of tissue-specific cofactors and downstream effector pathways.

#### Aging: KAT8 as a guardian of genome stability and a driver of epigenetic decline

KAT8 plays a dual role in aging, acting as a protector of genomic stability under normal conditions but contributing to age-related decline when its expression or activity is dysregulated. In the *zinc metallopeptidase STE24 (Zmpste24)*-deficient mouse model of premature aging (progeria), global reduction of H4K16ac impairs DDR, leading to accelerated senescence. Restoring H4K16ac levels either by MOF overexpression or by treatment with HDAC inhibitors ameliorates the premature aging phenotypes, including reduced DNA repair capacity and increased genomic instability.^145^

KAT8 maintains heterochromatin architecture by acetylating the chromatin remodeler remodeling and spacing factor 1 (RSF1) at K1050. In skin aging, depletion of the RNA demethylase FTO leads to increased m6A methylation of *KAT8* mRNA, which paradoxically enhances KAT8 expression via the reader protein IGF2BP3 (a similar FTO-m6A-IGF2BP3-KAT8 regulatory axis has been described in cerebellar development, “RNA epigenetic control of KAT8 expression in cerebellar development”).^132^ However, this elevated KAT8 is functionally uncoupled from RSF1 acetylation. These results in heterochromatin loss and accelerated skin aging phenotypes, such as reduced collagen and increased senescence markers.^20^

Under oxidative stress, KAT8 directly binds to and acetylates the promoters of antioxidant genes including *Prdx1*, *Gpx1*, and *Gpx4*, sustaining their expression to control intracellular ROS levels. KAT8 depletion downregulates these antioxidant genes leading to ROS accumulation and increased DNA double-strand breaks, which drive cellular senescence.^146^

Conversely, in skin aging, KAT8 participates in a homeostatic loop that counteracts age-related decline. Macrophages in aged skin produce lactate, which enters fibroblasts and induces KAT5-and KAT8-dependent histone H4 lysine 12 lactylation (H4K12la). This lactylation upregulates TGF β1 and TGF β3 expression, stimulating collagen synthesis in fibroblasts. The delactylase HDAC3 counteracts this modification, forming a regulatory loop that maintains skin homeostasis.^82^ Thus, KAT8 can both protect against aging (by maintaining DNA repair, heterochromatin, and antioxidant defense) and adapt to aging (by mediating metabolic epigenetic feedback), with its net effect depending on the balance of upstream signals and downstream effectors.

Whether in immunity, inflammation, fibrosis, or aging, the direction of KAT8 activity (protective versus pathogenic) is dictated by the same principles of complex assembly, substrate selection, metabolic cofactor availability, and cell type-specific signaling that govern its roles in cancer, metabolism, and neurology.

## Development and application of KAT8 inhibitors

As a core HAT of the MYST family, KAT8 is tightly linked to diverse human diseases through aberrant activation and has emerged as a prime drug discovery target. Progressive advances have been made in KAT8 inhibitor development, yielding a diversified portfolio including early non-selective inhibitors, novel highly selective compounds, natural products, and function-targeted molecules. Existing KAT8 inhibitors are summarized in Table 3.

**Table 3:** Inhibitors of KAT8

Name	Core mechanism/characteristics	Related disease/model	Advantages	Disadvantages	Reference
Aspirin/salicylate (SS)	inhibits multiple KATs; regulates differentiation and development	colon and prostate cancer (MUC1/EMT)	natural, low toxicity, clinically available	non-specific; difficult to target precisely	Fernandez et al.^127^
Anacardic acid (AA)	binds EX conformation, stabilizes inactive state	colon and prostate cancer, inflammatory diseases	simple structure, synthesizable, more potent than SS	non-specific; limited pharmacological and clinical data	Wapenaar et al.^24^; Ghizzoni et al.^147^
Gastrodin (GAS)	directly binds KAT8, reduces stability; inhibits H3K9la	rheumatoid arthritis models	natural, low toxicity, multi-pathway anti-inflammatory	very weak activity (KD = 413.72 μM); mechanism unclear; no clinical data	Dai et al.^18^
Capsaicin	upregulates KAT8, increases H4K16ac, induces G1 arrest	NSCLC, bladder cancer, glioma	unique upregulation mechanism; FDA-approved; multi-target antitumor	moderate oral bioavailability; GI irritation risk	Wang et al.^14^
Industry-developed inhibitors					
4-Amino-1-naphthol	binds free enzyme and acetylated intermediate; measures Ki	*in vitro* enzyme studies	differentiates binding affinity; precise activity assessment	lacks selectivity and cellular/*in vivo* data	Wapenaar et al.^148^
DC_M01_7.	competitively occupies H4 binding pocket; inhibits KAT8	colon cancer (HCT116 cells)	clear competitive mechanism; effective in cells	selectivity unvalidated; lacks IC50 and *in vivo* data	Zhang et al.^149^
CHI-KAT8i5	binds active site via H-bonds; inhibits acetyltransferase activity	esophageal squamous cell carcinoma (ESCC)	high potency and selectivity; effective *in vivo*	mechanism details pending; scope limited mainly to ESCC	Zhang et al.^12^
C646	provides key kinetic parameters for KAT8	*in vitro* enzymology	establishes screening standards; includes structure	non-specific; weak KAT8 targeting	Wapenaar et al.^24^
Compound 19 (optimized from C646 structure).	pyrrole replacement improves selectivity; reversible binding	NSCLC, AML cell models	high selectivity, reversible, low normal cell toxicity	lacks *in vivo* stability and PK data	Fiorentino et al.^150^
Compound 34 (optimized from C646 structure)	phenyl substitution enhances selectivity; induces apoptosis	NSCLC, AML cell models	more potent and selective than Cmpd 19; low toxicity	lacks *in vivo* stability and PK data	Fiorentino et al.^150^
MC4171	inhibits KAT8 activity; reduces H4K16ac	cerebellar developmental disorders	high selectivity; reverses acetylation abnormalities	may cross-inhibit MYST bamily; needs *in vivo* validation	Jiang et al.^132^
MG149	inhibits KAT8 acetyl-/lactyltransferase activity	various cancers, diabetes, lung injury models	clear mechanism; chemosensitizer	broad MYST inhibition; lacks clinical data	Xie et al.^56^; Xie et al.^72^; Miao et al.^83^; Guo et al.^130^; Chen et al.^151^; Chen et al.^152^^,^^153^
First-in-class (selective inhibitors of KAT8)	specifically inhibits KAT8 acetyltransferase activity	breast cancer, leukemia models	high selectivity vs. KAT5; induces arrest and apoptosis	effects in neurodegenerative models unstudied	Fiorentino et al.^150^
PF-9363 and analogs.	high doses inhibit KAT8, reduce H4K16Ac	triple-negative breast cancer	high oral bioavailability; multi-target potential	weak KAT8 targeting; high dose; off-target risk	Chen et al.^154^
KAT8-IN-1	inhibits autoacetylation; reduces H4K16Ac and cytokines	psoriasis (mouse model)	strong anti-inflammatory; oral/IP administration	needs long-term toxicity and PK studies	Xiang et al.^17^
Tumor-immunity targeting peptide					
Blocking peptide (2142–R8)	disrupts KAT8-IRF1 interaction; inhibits PD-L1	various solid tumor models	high specificity; targets protein–protein interaction	poor bioavailability; degradation and delivery challenges	Wu et al.^55^
Other targeted agents					
Gemcitabin	reduces KAT8 expression and H4K16ac; promotes apoptosis	multiple cancer models	clinically used; combinable with KAT8 targeting	low oral bioavailability; short half-life	Wang et al.^14^
Arsenic/arsenic trioxide	directly binds and inhibits KAT8	HeLa cells; myeloma; bladder cancer risk	clear binding; already used clinically	highly toxic; non-selective; damages normal tissues	Wang et al.^14^; Jo et al.^109^; Liu et al.^155^

### Development and screening of KAT8 inhibitors

#### Early non-selective inhibitors

Early inhibitors were mostly pan-lysine acetyltransferase (KAT) family inhibitors with limited selectivity. Solanacic acid (I) and its derivatives exhibited only mid-micromolar activity and lacked specificity.^150^ Salicylic acid (SS) inhibits multiple KATs including KAT8 and perturbs cellular differentiation.^127^ Anacardic acid (AA) stabilizes the inactive enzyme–acetyl complex (EX) conformation of KAT8 by binding the catalytic domain; its inhibition constant (Ki) and KAT8 enzymatic kinetic parameters (kcat 0.2–1.1 min^−1^) laid foundational data for subsequent studies.^24^ Across distinct enzymatic states, 4-amino-1-naphthol discriminates binding affinities underscoring the importance of kinetic characterization.^148^ Arsenic (As) directly binds the C2HC zinc-finger domain of KAT8 to inhibit activity,^109^^,^^155^ while DC_M01_7 acts by competitively occupying the histone H4-binding pocket.^149^

#### Novel highly selective inhibitors

To improve selectivity, N-phenyl-5-pyrazolone derivatives (compounds 19 and 34) were optimized based on the C646 scaffold and showed improved performance. Compound 34 exhibited an IC50 of 8.2 μM, with a KD of 2.04 μM for KAT8 binding measured by surface plasmon resonance (SPR). Both compounds specifically reduced cellular H4K16ac levels, displayed anti-proliferative activity (IC50 30–50 μM) in NSCLC and acute myeloid leukemia (AML) cells, and induced apoptosis and autophagy.^150^

#### Functional inhibitors and natural product inhibitors

MG149 is a widely used KAT8 functional inhibitor (Ki = 39 μM) that acts as a non-competitive inhibitor by stabilizing the enzyme’s inactive conformation.^156^ It shows therapeutic potential in multiple disease models: inhibiting osteogenic differentiation^151^; promoting YEATS4 degradation in bladder cancer to sensitize cells to cisplatin^56^; modulating pancreatic α-cell function to ameliorate type 2 diabetes^130^; reducing p53 acetylation to alleviate acute lung injury^152^; and inhibiting cyclin-dependent kinase 9 (CDK9) acetylation to regulate transcription.^153^

The natural product gastrodin (GAS) directly binds KAT8 (KD = 413.72 μM), reduces its stability, and suppresses H3K9 lactylation to alleviate inflammation in rheumatoid arthritis.^18^ Other natural flavonoids and the specialized small-molecule FI-3 also target KAT8, reversing aberrant acetylation in cancer cells and chemosensitizing tumor cells.^157^

#### Industrially developed inhibitors and alternative targeting strategies

The thiourea inhibitor PF-9363 (CTX-648) inhibits KAT8 and H4K16ac at high doses and exerts anti-proliferative effects in select cancer cell lines, although its multi-target nature requires cautious evaluation.^154^ Additionally, the blocking peptide 2142-R8 disrupts KAT8-IRF1 phase-separated condensates, represses PD-L1 expression, and enhances anti-tumor immunity.^55^ KAT8-IN-1 inhibits KAT8 autoacetylation and alleviates inflammation in psoriasis by interfering with KAT8-AP-1 interactions.^17^ Several chemotherapeutic agents (e.g., gemcitabine, arsenic trioxide) and non-coding RNAs also modulate KAT8 expression or activity.^14^

### Mechanisms of action of KAT8 inhibitors

Distinct inhibitors act through diverse mechanisms: stabilizing the catalytically inactive conformation (e.g., AA, MG149)^24^^,^^156^; competitively binding substrate pockets (e.g., DC_M01_7)^149^; impairing protein stability (e.g., GAS)^18^; or disrupting protein-protein interactions (e.g., KAT8-IN-1).^17^

Downstream effects are broad: in inflammation, inhibitors block NLRP3 activation and interleukin-6 (IL-6) secretion by suppressing p53 acetylation or histone lactylation^18^^,^^152^; in cancer, they induce apoptosis and autophagy, or exert anti-proliferative effects by inhibiting aurora kinase B (AURKB) acetylation, H4K16ac, and other oncogenic modifications^72^^,^^150^^,^^154^; they also block KAT8 lactyltransferase activity, abrogating tumor metabolic reprogramming driven by lactylation of substrates such as eEF1A2.^83^

### Therapeutic potential of kat8 inhibitors in diseases

#### Cancer therapy

KAT8 inhibitors show significant potential in reversing tumor drug resistance and targeting oncogenic signaling pathways. KAT8-mediated H4K16ac is central to HDAC inhibitor-mediated sensitization to topoisomerase II inhibitors, and targeting this axis can overcome therapeutic resistance.^158^ KAT8 has been validated as a key therapeutic target in KIRC, GBM (via the KAT8-EGFR axis), breast cancer (via the KAT8-AURKB axis), and triple-negative breast cancer.^40^^,^^72^^,^^107^^,^^154^

To realize the therapeutic promise of KAT8 inhibition, the field must navigate a paradigm shift from broad enzymatic suppression to contextually guided intervention. This necessity arises directly from the functional heterogeneity cataloged in Table 2. As discussed in “tumor-suppressive functions of KAT8: mechanisms and contexts” KAT8 exhibits context-dependent duality where it often acts as an oncogene but paradoxically serves as a tumor suppressor in specific settings. This substrate-dependent plasticity implies that a pan-inhibitor might simultaneously block oncogenic pathways like KAT8/c-Myc in ESCC while undermining tumor-suppressive mechanisms like KAT8/FASN in HCC, potentially leading to unpredictable or adverse clinical outcomes.

Current inhibitor development reflects this structural challenge. While selective molecules like compound 34 show improved specificity over early pan-KAT inhibitors,^150^ they target the shared catalytic domain. Consequently, even selective inhibitors risk disrupting both oncogenic signaling and fundamental homeostatic functions, such as DDR via p53 acetylation or mitophagy regulation via PINK1 modulation.^40^^,^^134^ The limited *in vivo* efficacy and lack of systemic safety data for current leads, as noted in Table 3, likely stem from this inability to dissect these overlapping functional roles. Overcoming this impasse requires the development of precision targeting strategies anchored in multifactorial biomarker frameworks. We propose that patient stratification cannot rely on KAT8 expression alone but must integrate catalytic output and substrate specificity. Tumors exhibiting “KAT8 oncogene addiction” might be defined by high KAT8 expression concurrent with elevated H4K16ac at oncogene promoters (as seen in NSCLC^40^) and active non-histone lactylation driving metabolic reprogramming (such as eEF1A2 lactylation in CRC).^83^ These patients could be candidates for catalytic inhibitor monotherapy or combinations with DNA-damaging agents. Conversely, tumors where KAT8 maintains tumor suppressor gene expression (like TMS1 in breast cancer)^83^ or acts as a metabolic brake (like FASN in HCC)^74^ should be excluded from KAT8 inhibition trials to avoid exacerbating genomic instability. Furthermore, in tumors driven by specific protein-protein interactions rather than broad catalytic activity (like the KAT8-IRF1 condensate driving PD-L1 expression),^55^ future strategies might prioritize targeted disruptors like the blocking peptide 2142-R8 over traditional small molecules. Such a multidimensional classification system is a prerequisite for translating KAT8 biology from a catalog of contradictory mechanisms into actionable, safe clinical protocols.

## Non-neoplastic diseases

In inflammatory and metabolic disorders, MG149, GAS, and KAT8-IN-1 exhibit efficacy in models of acute lung injury, rheumatoid arthritis, and psoriasis, respectively.^17^^,^^18^^,^^152^ In neurological and developmental diseases, modulating KAT8/NSL complex function holds promise for correcting mitophagic defects in Parkinson’s disease^134^; the KAT8 inhibitor MC4171 partially rescues cerebellar hypoplasia caused by aberrant FTO-m6A signaling^132^; in placental developmental disorders, elevating H4K16ac with SIRT1 inhibitors represents a potential therapeutic strategy.^96^

### Challenges and future directions

Despite recent progress in KAT8 inhibitor development, several major challenges impede their translation into clinical practice. First, current inhibitors lack specificity. Most of them target broad MYST or KAT family members. They do not have high selectivity for KAT8. Second, our mechanistic understanding is incomplete. The regulatory networks of KAT8 depend on the context of different diseases. In some cancers, KAT8 can act as both an oncogene and a tumor suppressor. How this dual role is controlled is still poorly defined. Third, the properties of existing compounds are suboptimal. They need better potency, improved *in vivo* stability, and higher bioavailability. Long-term systemic safety data are also limited.

Future directions will focus on several areas. One is structure-based rational design. Researchers can exploit the unique features of the KAT8 active pocket. This can help develop highly selective inhibitors with minimal off-target effects on other MYST family members. Another direction is mechanistic dissection and precision medicine. We need to clarify context-specific KAT8 regulatory networks. We should also explore crosstalk with metabolic reprogramming, for example lactylation. Combination therapies can then be developed. A third direction is pharmacokinetic optimization and biomarker development. We need to improve the pharmacokinetic properties of candidate compounds. We should also establish biomarker panels based on histone modifications. Examples include H4K16ac and H3K9la. These biomarkers can be used for therapeutic monitoring and patient stratification.

#### Summary

KAT8 inhibitor development has evolved from broad-spectrum tool compounds to highly selective therapeutic candidates. Despite remaining challenges in specificity, potency, and mechanistic clarity, advances in multidisciplinary technologies position KAT8 targeting as a promising epigenetic therapeutic strategy for cancer, inflammation, neurodegenerative diseases, and other disorders.

## Conclusion

KAT8, a pivotal member of the MYST family, has evolved far beyond its canonical role in X chromosome dosage compensation to become a central epigenetic hub with a complex regulatory network and highly multifunctional activities. This review systematically demonstrates that KAT8, primarily through the NSL and MSL complexes, catalyzes H4K5ac, H4K8ac, and H4K16ac and modifies non-histone substrates to regulate diverse cellular processes. Its functions are precisely targeted and partitioned through the MSL and NSL complexes, and tightly regulated by intrinsic post-translational modifications (e.g., autoacetylation, phosphorylation) and upstream signaling pathways, highlighting the importance of complex context for KAT8 substrate specificity and function.

In disease contexts, KAT8 exhibits striking context dependency and acts as a “double-edged sword.” In most cancers, its overexpression or aberrant activation drives tumor progression by promoting proliferation, invasion, metabolic reprogramming, and therapeutic resistance. Concurrently, KAT8 contributes to the pathophysiology of neurodegenerative diseases (via mitophagy and chromatin homeostasis), immune-inflammatory disorders (via regulation of key immune factors), and aging (via genome stability maintenance). These findings establish KAT8 as a common molecular node linking multiple major human diseases. Recent studies have refined the understanding of KAT8 complex-dependent substrate specificity and revealed context-dependent roles of H4K16ac in transcription versus replication timing.^22^^,^^23^ Although preliminary progress has been made in KAT8 inhibitor development, its translational potential remains largely untapped. Future research must dissect the precise functional networks of KAT8 in tissue- and disease-specific microenvironments, and develop novel targeted agents with high selectivity and favorable pharmacokinetic properties.

## Future perspectives

The field of KAT8 research is entering a transformative phase. A key priority is to understand how KAT8 switches between the MSL and NSL complexes in response to metabolic cues, DNA damage, or cytokines. Mapping the full substrate landscape of KAT8, including non-canonical acylations such as lactylation, remains essential. Cross-talk between KAT8-mediated acylations and other modifications like methylation and ubiquitination should be explored to build an integrated regulatory network. The context-dependent dual roles of KAT8 require further clarification. A better understanding of why KAT8 acts as an oncogene in some cancers but as a tumor suppressor in others will guide rational therapy design. Measuring KAT8 activity, H4K16ac levels, or specific acylation marks in tissues or blood may serve as biomarkers for diagnosis and treatment monitoring. Expanding studies into AD and schizophrenia could reveal new therapeutic opportunities. One major unanswered question is whether KAT8-catalyzed acylations such as propionylation and lactylation are passive metabolic byproducts or active signaling events. Decoding this potential “acylation code” will require systematic genome-wide mapping and analysis of cross-talk between different acyl marks. Advances in drug development are critical. Structure-based design can yield highly selective KAT8 inhibitors with fewer off-target effects. PROTAC-mediated protein degradation offers a promising alternative to conventional inhibition. Combining KAT8 inhibitors with chemotherapy, immunotherapy, or other epigenetic drugs may also overcome resistance and improve efficacy.

## Data and code availability

The authors have nothing to report.

## Acknowledgments

Figures 1, 2, 3, 4, 5, 6, 7, and 8 in this review were created with BioRender.com and BioGDP.com.^159^ Nomenclature for genes and proteins follows established species-specific conventions: for human genes/proteins, *KAT8 (gene*, *italic)* and KAT8 (protein); for mouse, *Kat8 (gene*, *italic)* and MOF (protein); for Drosophila, *MOF (gene*, *italic)* and MOF (protein). Complex abbreviations (MSL, NSL) are defined in Table S2.

This work was supported by the Foundation of Shandong Natural Science (ZR2023MH080, ZR2019BH007), the 10.13039/501100001809National Natural Science Foundation of China (82304109), the Youth Foundation of Shandong Natural Science Foundation (ZR2021QH298).

## Author contributions

G.P., conceptualization, supervision, and writing – reviewing and editing; W.Z. and R.G., data curation, methodology, software, and writing – original draft preparation; R.G., visualization and investigation. All authors have read and approved the final manuscript.

## Declaration of interests

The authors declare no conflicts of interest.

## Declaration of generative AI and AI-assisted technologies in the writing process

During the preparation of this work the authors used DeepSeek for grammatical and spelling checks. The use of AI was strictly confined to language enhancement tasks such as translation and grammar checking. After using this tool, the authors reviewed and edited the content as needed and take full responsibility for the content of the publication.

## Supplemental information

Table S1. Core MYST family members and their cross-species ortholog nomenclature

Table S2. Nomenclature and naming convention for KAT8/MOF-related genes, proteins, and complexes across species
