# Supplementary material for: KAT8-mediated epigenetic modifications: Physiological functions, heterogeneity in disease, and advances in targeted development
Source: iScience. 2026 Jul 10;29(8):116680. doi: 10.1016/j.isci.2026.116680 (PMC13380446; doi:10.1016/j.isci.2026.116680)
Supplement: Table S1. Core MYST family members and their cross-species ortholog nomenclature [file mmc1.pdf]

iScience, Volume *29*

Supplemental information

KAT8-mediated epigenetic modifications:
Physiological functions, heterogeneity
in disease, and advances in targeted development

Zengjin Wang, Guanghui Ren, and Peng Gao

Supplementary Table I Core MYST family members and their cross-species ortholog nomenclature

Core MYST member	Human (*Homo sapiens*)	Mouse (*Mus musculus*)	Fruit fly (*Drosophila melanogaster*)	Budding yeast (*Saccharomyces cerevisiae*)	Thale cress (*Arabidopsis thaliana*)
**MOZ/MORF**	KAT6A(MOZ,MYST3)<br>KAT6B(MORF,MYST4)	Kat6a (Myst3)<br>Kat6b (Myst4)	Enok (ortholog)	Sas3	N/A
**Tip60**	KAT5 (Tip60, HTATIP)	Kat5 (Tip60)	Tip60	Esa1	N/A
**HBO1**	KAT7 (HBO1, MYST2)	Kat7 (Hbo1, Myst2)	CG1894 (putative)	No clear ortholog	N/A
**MOF**	KAT8 (MOF, MYST1)	Kat8 (Mof)	MOF	Sas2	N/A
**Plant-specific**	N/A	N/A	N/A	N/A	HAM1 (HAM1)<br>HAM2 (HAM2)

N/A indicates not applicable.
