# Supplementary material for: KAT8-mediated epigenetic modifications: Physiological functions, heterogeneity in disease, and advances in targeted development
Source: iScience. 2026 Jul 10;29(8):116680. doi: 10.1016/j.isci.2026.116680 (PMC13380446; doi:10.1016/j.isci.2026.116680)
Supplement: Table S2. Nomenclature and naming convention for KAT8/MOF-related genes, proteins, and complexes across species [file mmc2.pdf]

Supplementary Table II Nomenclature and naming convention for KAT8/MOF-related genes, proteins, and complexes across species

Term	Species	Type	Description & Official/Preferred Name	Usage in the Manuscript & Recommended Consistency
**KAT8**	Human (Homo sapiens)	Gene Symbol	Official HUGO Gene Nomenclature Committee (HGNC) gene name for lysine acetyltransferase 8.	Primary preferred gene name in human contexts (e.g., "KAT8 is highly expressed…").
**MOF**	Human (Homo sapiens)	Protein Alias	Most common protein alias for KAT8, derived from "males-absent-on-the-first".	Acceptable as protein name (e.g., "MOF protein", "MOF acetylation"). Should not be used as human gene symbol.
**MYST1**	Human (Homo sapiens)	Protein Alias	Alternative protein alias, indicating its membership in the MYST family.	Less preferred; use "KAT8" or "MOF" for clarity.
**Kat8**	Mouse (Mus musculus)	Gene Symbol	Official mouse gene name; capital K only, rest lower case, italicized.	Preferred gene name in mouse contexts (e.g., "Kat8 knockout mice").
**Mof**	Mouse (Mus musculus)	Protein Name	Standard mouse protein name; only first letter capitalized, not italicized.	Preferred protein name in mouse contexts (e.g., "MOF-mediated acetylation").
**MOF**	Drosophila (Drosophila melanogaster)	Gene & Protein Name	Original name identified in flies; conventionally capitalized and italicized for gene.	Preferred term for both gene and protein in Drosophila (e.g., "MOF mutation").
**MSL complex**	Broad (Conserved)	Protein Complex	Male-specific lethal complex. Core catalytic subunit is KAT8/MOF.	Standard acronym. Spell out on first use. Use consistently across species (e.g., "the MSL complex").
**NSL complex**	Broad (Conserved)	Protein Complex	Non-specific lethal complex. Core catalytic subunit is KAT8/MOF.	Standard acronym. Spell out on first use. Use consistently across species (e.g., "the NSL complex").

**MSL1v1 complex or KAT8‑KANSL1**	Broad (Conserved)	Protein/Complex Component	MSL1v1 is actually the same protein as KANSL1 (KAT8 regulatory NSL complex subunit 1)	Use as given. Clarify it is distinct from the canonical NSL complex in subunit composition and substrate specificity.
